# Lithiophilic Current Collector Without Interfacial Penalty in Zero‐Excess Lithium Metal Batteries

**DOI:** 10.1002/advs.202519303

**Published:** 2026-03-28

**Authors:** Jiyeon Seo, Sangseob Lee, Seojin Jeon, Minhong Lim, Hyegang Koo, Seung‐Tae Hong, Woosun Jang, Aloysius Soon, Hongkyung Lee

**Affiliations:** ^1^ Department of Materials Science and Engineering Yonsei University Seoul Republic of Korea; ^2^ Department of Energy Science and Engineering Daegu Gyeongbuk Institute of Science and Technology (DGIST) Daegu Republic of Korea; ^3^ Department of Battery Engineering Yonsei University Seoul Republic of Korea; ^4^ Integrated Science and Engineering Division Yonsei University Incheon Republic of Korea

**Keywords:** bilayer coating, current collectors, lithiophilicity, solid electrolyte interphase, zero‐excess Li metal batteries

## Abstract

Highly reversible lithium (Li) plating/stripping in zero‐excess Li metal batteries (ZE‐LMBs) demands lithiophilic current collectors to suppress Li dendrite formation and Li pulverization. Although Li‐alloyable metals have been recognized as lithiophilic substrates, their structural and interfacial stability over cycling are still poorly understood. Here, we present a bimetallic lithiophilic current collector through sequential coatings of platinum (Pt) and silver (Ag). Experimental and computational studies reveal that Ag facilitates uniform Li nucleation and seamless solid electrolyte interphase (SEI) formation owing to the low Li diffusion barrier and strong anion adsorption, whereas Pt maintains lithiophilicity and structural integrity. Leveraging this complementarity, the Ag‐outer/Pt‐inner bilayer (Ag/Pt@Cu) achieves superior cycling stability through location‐specific functional decoupling: the outer Ag layer ensures uniform Li deposition and robust SEI formation, whereas the inner Pt layer supports long‐term lithiophilicity, thereby outperforming the reversed configuration (Pt/Ag@Cu). Given that the structural robustness of lithiophilic coatings is essential for enhancing the cycling performance of ZE‐LMBs, this study provides a versatile design framework for multi‐component, multi‐layer architectures, enabling the rational engineering of structurally resilient, lithiophilic current collectors.

## Graphical abstract

Bilayer lithiophilic coatings are introduced to overcome the structural fragility of conventional single‐layer current collectors in zero‐excess Li‐metal batteries (ZE‐LMBs). By integrating density functional theory with electrochemical analyses, the Ag‐outer/Pt‐inner configuration enables uniform Li deposition, robust SEI formation, and persistent lithiophilicity, delivering remarkably stable and long‐lasting cycling performance in Cu||NMC811 full cells.

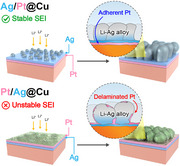

## 1 Introduction

Zero‐excess Li‐metal batteries (ZE‐LMBs), also known as anode‐free batteries, have emerged as a promising next‐generation battery architecture due to their potential for ultrahigh energy density, simplified cell design, and reduced material and manufacturing costs [1, 2, 3, 4, 5, 6, 7, 8, 9, 10]. Unlike intercalation hosts such as graphite or silicon used in today's Li‐ion batteries (LIBs), ZE‐LMBs do not require a host material, as Li^+^ is directly plated onto the current collector during charging. Removing the graphite anode, which accounts for approximately 28% of the cell's weight and 46% of its thickness, enables ZE‐LMBs to achieve theoretical gains of 38.5% in specific energy and 85.5% in volumetric energy density [10, 11, 12, 13]. Moreover, the elimination of anode slurry coating and highly reactive Li metal simplifies the manufacturing process, potentially reducing production costs by 18% compared to current LIBs and by 50% compared to conventional LMBs [6].

Nonetheless, ZE‐LMBs suffer from serious safety risks and shorter cycle life, sharing key challenges common to LMBs [3, 6, 11, 14, 15, 16, 17, 18, 19]. Spatially non‐uniform Li plating and stripping and the high reactivity of Li are directly linked to poor interfacial stability, leading to the fatal growth of Li dendrites and low Coulombic efficiency (CE). Given that ZE‐LMBs contain no excess Li, preserving available Li is of utmost importance. Li inventory loss is primarily caused by two factors: (1) the ‘dead’ Li formation due to electrical isolation of Li filaments during random Li stripping, and (2) extensive formation of the solid‐electrolyte interphase (SEI) because of its inherent structural and chemical instability [20]. These detrimental processes recur with each cycle, progressively depleting active Li and degrading cell capacity. Thus, regulations of Li plating, stripping, and robust SEI buildup should be simultaneously achieved for long‐term stable cycling of ZE‐LMBs.

As a key component, the current collector serves as the reaction site for storing Li ions via electrodeposition. Lithiophilicity has catalyzed extensive research to design current collectors that suppress sporadic, dendritic Li deposition and detrimental Li pulverization [21, 22, 23, 24]. Several Li‐alloyable metals such as silver (Ag), gold (Au), zinc (Zn), magnesium (Mg), and platinum (Pt) have been proposed to lower the Li nucleation barrier and promote uniform Li deposition [22, 25, 26, 27, 28, 29, 30]. Furthermore, recent advances have introduced composite Li metal anodes and 3D host architectures, such as coralloid Ag‐coated carbon fibers, Ag‐carbon composite interlayers, and CoF_2_@C hollow spheres, to effectively accommodate Li volume expansion and reduce local current density [31, 32, 33]. Although these strategies demonstrate impressive stability, they often involve complex fabrication processes or introduce additional inactive weight and volume (i.e., volumetric penalty), which can compromise the energy density of ZE‐LMBs. Despite many efforts devoted to achieving optimal lithiophilicity, most studies have often overlooked how the original surface evolves during cycling and how it influences SEI chemistry. Li‐alloy metals can form either solid‐solution (SS) or intermetallic (IM) phase [29, 30], both of which can affect the persistence of lithiophilicity and interfacial stability. For instance, both Ag and Pt effectively suppress dendritic Li growth but exhibit contrasting interfacial behaviors. Ag, which forms a SS phase with Li, struggles to sustain surface lithiophilicity due to extensive interatomic mixing with bulk Li and the accumulation of electrochemically inactive Ag residues (“dead” Ag) [34, 35]. In contrast, Pt forms an IM phase with Li and slows interatomic diffusion, thereby preserving its original structure. However, the substrate‐dependent SEI chemistry of Pt requires further investigation [34].

Motivated by these distinct interfacial behaviors, this work proposes a bimetallic approach using bilayer coating of Li‐alloying metals for sustainable lithiophilicity without compromise in structural stability and SEI formation. The bilayer coating includes Pt inner layer to retain structural stability and Ag outer layer to secure interfacial stability, thereby synergistically improving the cycling performance of ZE‐LMBs, even compared to bare Cu and the reverse coating. Electrochemical results and first‐principles calculations reveal that lithiophilicity alone does not guarantee improved cycling performance; rather, the persistence of lithiophilic properties and their compatibility with the electrolyte are key to ensuring the long‐term stability of ZE‐LMBs.

## 2 Results and Discussion

**FIGURE 1:**
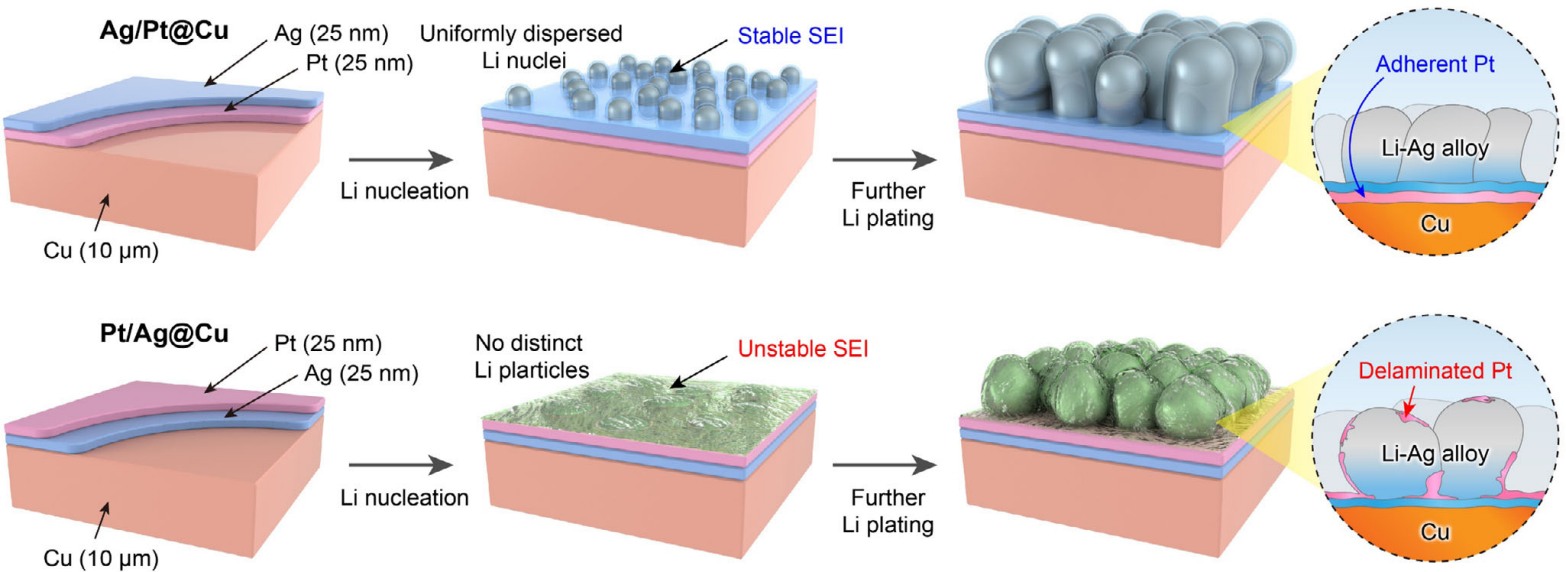
Scheme of Li plating on Ag/Pt@Cu and Pt/Ag@Cu. Ag/Pt@Cu enables stable SEI formation and uniform Li nucleation, leading to dendrite‐less, dense Li deposition at the outer Ag surface, whereas the inner Pt layer remains adherent to Cu current collector. In contrast, SEI buildup is deterred on Pt/Ag@Cu, leading to the growth of round‐shaped Li deposits and the formation of Li–Ag alloy that delaminates the outer Pt layer.

While Li‐alloyable metals are widely recognized as lithiophilic materials that suppress Li dendrite growth, their dynamic interaction with electroplated Li during cycling complicates the persistence of lithiophilicity and stable SEI buildup. Our earlier work revealed that, while both Ag and Pt effectively inhibit dendritic Li plating, their distinct alloying behaviors with Li (i.e., SS and IM) result in substantial disparities in structural stability and SEI formation [29, 34]. These complementary strengths and weaknesses motivated us to adopt a bimetallic approach. Interfacial processes are governed at the outermost surface of the bimetallic substrate in contact with plated Li, necessitating location‐specific functional allocation. To address this, we introduced the concept of functional decoupling through a bilayer metallic coating, fabricated by sequential coatings of Pt and Ag on the current collector (Figure 1). These configurations are hereafter denoted as Ag/Pt@Cu and Pt/Ag@Cu, where the notation ‘X/Y@Cu’ explicitly defines the outer X layer in contact with the electrolyte and the inner Y layer in contact with Cu substrate.

Positioning Ag at the outer layer (Ag/Pt@Cu) promotes favorable SEI formation but suffers from poor structural stability due to extensive interatomic diffusion into bulk Li. In contrast, the Pt surface of Pt/Ag@Cu is less effective in facilitating robust SEI buildup but maintains high structural stability through intermetallic compound formation. While SEM images revealed comparable surface morphologies across all samples, EDS mapping and atomic ratios confirmed clear distinctions consistent with the intended coating sequence (Figure S2). Notably, both Ag/Pt@Cu and Pt/Ag@Cu exhibited an outer layer metal content of ≥75%, indicating that each surface is predominantly composed of a single element. In the electrochemical responses, cyclic voltammetry (CV) reveals that both metals in bilayer coatings participated in Li‐alloy reactions prior to Li deposition [34, 36]. However, the pronounced anodic peak of Li─Ag in Ag/Pt@Cu indicates that the surface composition is clearly determined by the coating sequence (Figure S3). This validates the bilayer‐structured bimetallic current collectors and provides a solid basis for comparing interfacial and structural stability as a function of layer positioning.

**FIGURE 2:**
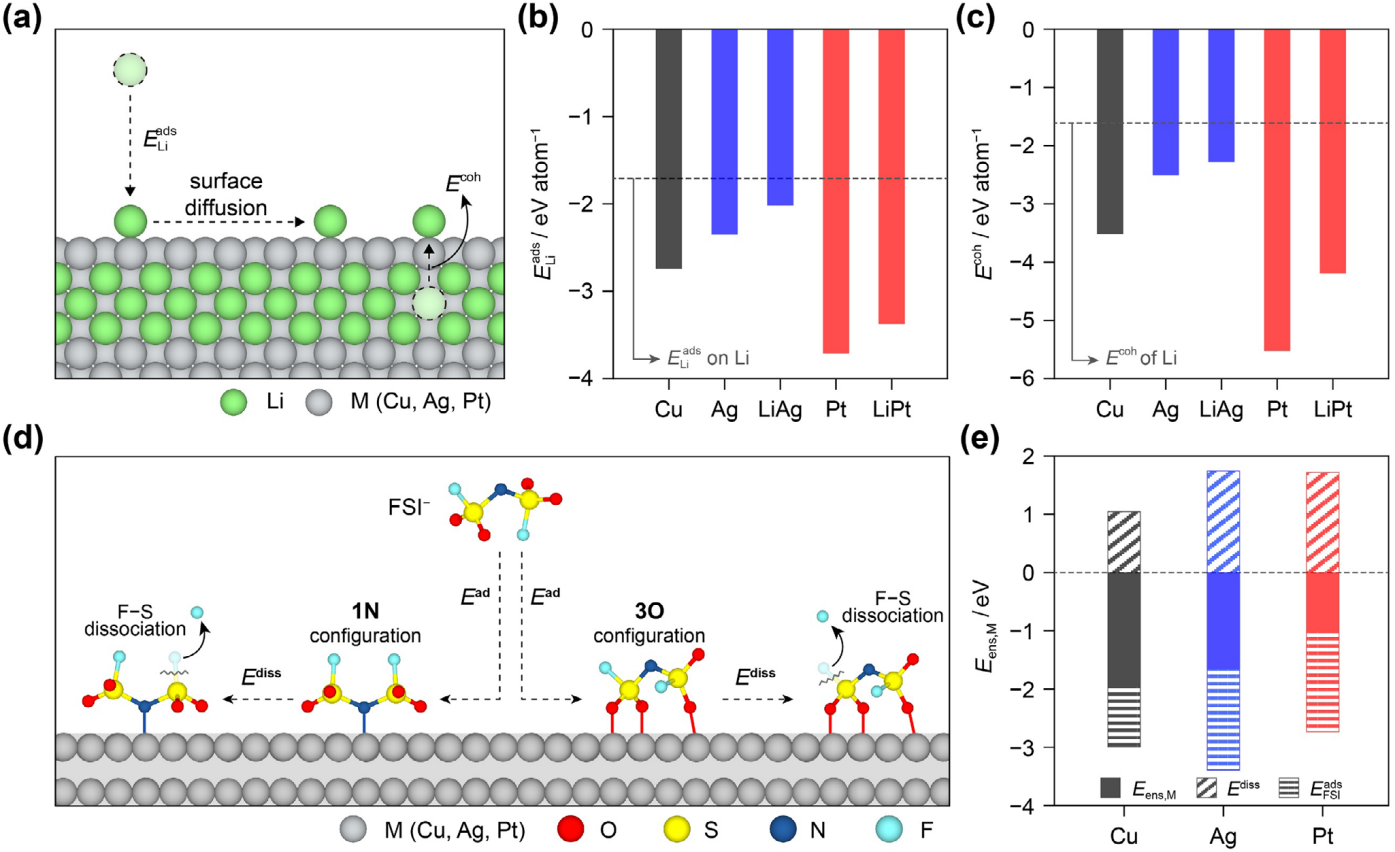
Computational analysis of Li plating and anion decomposition behaviors. (a) Scheme for interfacial behaviors of Li occurring on and within metal surfaces and subsurfaces. (b) DFT‐calculated Li adsorption energies ($E_{\mathrm{Li}}^{\mathrm{ads}}$) on various metal surfaces, (c) cohesive energies (*E* ^coh^) of bulk metals and alloy structures. (d) Scheme for FSI^−^ anion adsorption and S─F bond cleavage on metal surfaces, considering two configurations: nitrogen‐approaching (1N) and three‐oxygen coordination (3O). (e) Ensemble‐averaged energy (*E*_ens,M_) incorporating both FSI^−^ adsorption energy $(E_{\mathrm{FSI}}^{\mathrm{ads}}$, horizontally shaded) and S─F bond dissociation energy (*E* ^diss^, diagonally shaded) on metal surfaces.

First‐principles density‐ functional theory (DFT) calculations were performed to elucidate Li plating nature at pristine metals (Cu, Ag, and Pt), and alloys (Li─Ag and Li─Pt) (Figure 2a). Based on previous reports [37, 38, 39], the 1:1 stoichiometric phase of Li─Ag (BCC) and Li─Pt (HCP) alloys were selected, as they exhibit the lowest formation energies relative to their bulk reference states. The lowest‐energy surface slab structures were determined accordingly (Table S1), and Li adsorption energies ($E_{\mathrm{Li}}^{\mathrm{ads}}$) were calculated for various high‐symmetry sites (Table S2). As shown in Figure 2b, pristine Pt exhibits stronger Li binding ($E_{\mathrm{Li}}^{\mathrm{ads}}$= −3.71 eV) than Ag (−2.35 eV). This trend extends to alloy surfaces, implying that $E_{\mathrm{Li}}^{\mathrm{ads}}$ is primarily governed by the elemental species at the surface. Strong binding anchors Li at energetically preferred sites, reducing nucleation density and hindering surface redistribution. In contrast, relatively weaker adsorption (yet still thermodynamically favorable) enhances adatom mobility, facilitating widespread nucleation and more conformal deposition. Assessing bulk cohesive energies (*E* ^coh^) offers further insight into the structural stability of lithiophilic coatings (Figure 2c). While both Li─Ag and Li─Pt alloys are thermodynamically stable with negative $E_{\mathrm{Li}}^{\mathrm{coh}}$ values, a weaker cohesion of the Li─Ag alloy (−2.28 eV atom^−1^) implies that Ag atoms can more readily diffuse into bulk Li than Pt atoms in the Li─Pt alloy (−4.19 eV atom^−1^). This interatomic mixing of Ag with Li deposits can compromise the structural stability of the original coating and lead to Ag depletion over prolonged cycling [34, 35].

Different metal surfaces can lead to distinct SEI formation scenarios. Since anion‐derived SEI is dominant in localized high‐concentration electrolyte (LHCE), the adsorption energies of FSI^−^ anion ($E_{\mathrm{FSI}}^{\mathrm{ads}}$) on Pt, Ag, and Cu surfaces were primarily examined. Given the minimal conformation energy of the FSI^−^, $E_{\mathrm{FSI}}^{\mathrm{ads}}$ values were comparatively calculated for multiple binding motifs (Figure S4). Figure 2d depicts two primary adsorption configurations of the FSI^−^ anion: (i) a nitrogen‐approaching (1N) geometry and (ii) a three‐oxygen bonding geometry (3O). Compared to moderate FSI^−^ adsorption (−3.06 eV) at the Cu (3O configuration), Ag exhibited the stronger binding (−3.47 eV) in the 1N geometry. In contrast, Pt showed the weaker interaction with FSI^−^ (−2.79 eV), also in the 1N configuration. The S─F bond dissociation energy (*E* ^diss^) was calculated as a descriptor of LiF formation. Two asymmetric fluorine sites in the 3O geometry (3O‐1 and 3O‐2) were separately considered (Figure S5). Ag and Pt exhibited comparable *E* ^diss^ values (*∼*1.7 eV), whereas Cu required a lower dissociation energy (*∼*1.1 eV). As a unified descriptor of SEI formability, we calculated ensemble‐averaged energies (*E*_ens,M_) using classical Boltzmann statistics, which incorporate both adsorption and dissociation contributions across the 1N and 3O configurations (Figure 2e). As a result, Cu (*E*_ens,M_ = −1.99 eV) and Ag (−1.69 eV) were found to outperform Pt (−1.03 eV). This ensemble‐averaged descriptor suggests that the Pt surface is relatively less effective at inducing anion‐derived SEI formation.

To sum up, our DFT study decodes a mechanistic framework of Li plating morphology and SEI formation: positioning the Ag layer at the outer layer is beneficial to uniform Li nucleation and anion‐derived SEI buildup as intended owing to moderate Li binding and strong anion adsorption. In contrast, the Pt layer underneath retains sustainable lithiophilicity, contributing structural and interfacial support. This layered positioning (Ag/Pt@Cu) leverages Ag for initiating conformal Li deposition and stable SEI formation, while Pt serves as a long‐lasting interfacial support even after “dead Ag” formation or Ag depletion. For Pt/Ag@Cu, however, cycling stability can be degraded due to the limited SEI‐forming capability of the Pt surface.

**FIGURE 3:**
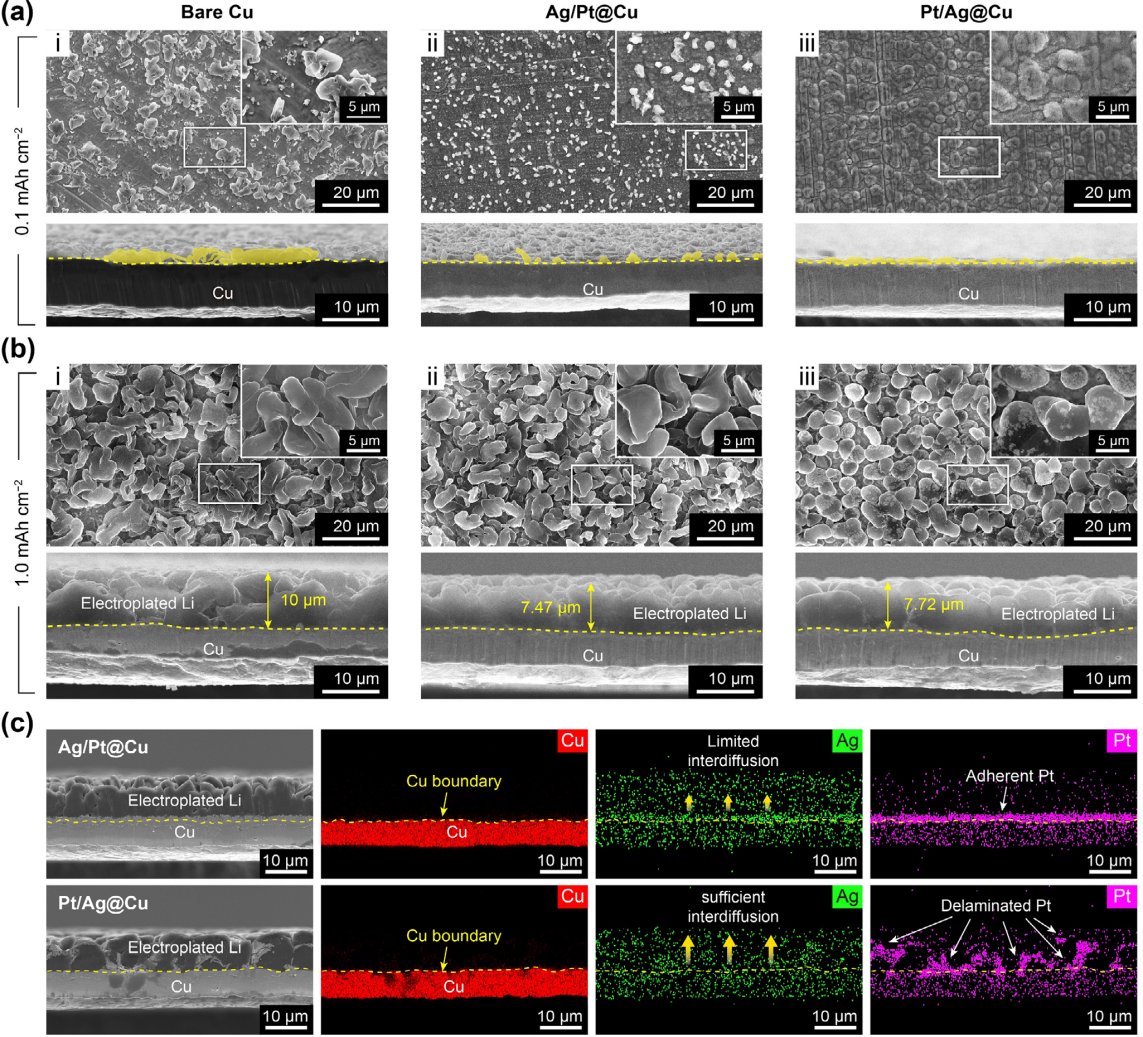
Substrate‐dependent Li plating behaviors and structural stability of bilayer coatings. SEM images obtained at the Li plating capacity of (a) 0.1 and (b) 1.0 mAh cm^−2^ for all samples (Inset: magnified view). (c) Cross‐section SEM images and EDS mapping for Ag/Pt@Cu and Pt/Ag@Cu after Li plating (1.0 mAh cm^−2^) at 0.5 mA cm^−2^.

To validate the DFT studies, we first investigated electrochemical behavior and Li plating morphology. Chronoamperometry (CA) was employed to analyze the nucleation and growth modes (Figure S6a). For a fair comparison, a sputtered Cu sample (Cu@Cu) was prepared to exclude the effect of native surface oxides on bare Cu. Compared with theoretical Scharifker–Hill profiles, the dimensionless plots show that bare Cu (and Cu@Cu) likely followed a 3D progressive nucleation mode, and a rapid current decay after the maximum point indicates diffusion‐limited growth with relatively few active nucleation sites [40, 41, 42, 43]. Indeed, SEM images taken at an early stage of Li plating (∼0.1 mAh cm^−2^) reveal that bare Cu formed sporadic, irregular Li nuclei, indicative of uncontrolled, heterogeneous nucleation (Figure 3a‐i). The bilayer (Ag/Pt@Cu and Pt/Ag@Cu) and single‐layer (Pt@Cu and Ag@Cu) coatings consistently showed 3D instantaneous nucleation (Figure S6b). SEM images confirmed that Ag/Pt@Cu enabled Ag‐assisted widespread Li nucleation and more conformal deposition (Figure 3a‐ii), consistent with our DFT calculations. Notably, no distinct Li particles were observed on the Pt/Ag@Cu surface at the very initial stage (Figure 3a‐iii). Instead, most Li was seemingly expended in alloying reactions with Ag and subsurface nucleation, rather than depositing directly on the Pt surface. As confirmed by CV (Figure S3), Li partly participated in Ag alloying even when positioned beneath Pt. Given the distinct volume changes associated with Li alloying in Ag and Pt [30, 44, 45, 46, 47, 48, 49], this mismatch imposes mechanical stress on the Pt layer, generating microcracks and ultimately collapsing the bilayer structure.

After further Li plating (1.0 mAh cm^−2^), Li deposits on bare Cu remained highly irregular and loosely packed (Figure 3b‐i), whereas both Ag/Pt@Cu and Pt/Ag@Cu enabled more compact Li deposits, achieving plating densities exceeding ∼60%. While Ag/Pt@Cu partially regulated Li deposition into a relatively uniform morphology (Figure 3b‐ii), Pt/Ag@Cu exhibited even more uniform and round‐shaped Li particles, likely due to the strong Li affinity of Pt (Figure 3b‐iii). Nonetheless, compared with the smooth surfaces of Li deposits on bare Cu and Ag/Pt@Cu, the Li surface plated on the Pt/Ag@Cu was locally contaminated by fractured Pt residues, as confirmed by EDS mapping (Figure S7). Cross‐sectional EDS analyses clearly elucidate the structural stability of the bilayer coatings (Figure 3c). The Ag in Ag/Pt@Cu readily alloyed and dispersed with plated Li, while the inner Pt layer remained adherent and structurally intact, thereby preserving the bilayer configuration. In Pt/Ag@Cu, however, Li penetrated beneath the Pt layer and alloyed with the inner Ag layer, which led to partial delamination of Pt and its irregular dispersion within the Li deposits. This corroborates the subsurface nucleation and Pt debris detected at the Li surface. Notably, single‐layer Pt coating (Pt@Cu) also showed partial delamination, highlighting that bimetallic bilayer (Ag/Pt@Cu) coating is more effective in enhancing structural stability (Figure S8). Positioning Ag at outer layer serves as a buffer that offsets the structural fragility of Pt during Li alloying, preserving interfacial lithiophilicity. Therefore, optimizing surface lithiophilicity alone is insufficient; more importantly, structural stability must be carefully engineered through appropriate coating sequences in bilayer (or multilayer) designs.

**FIGURE 4:**
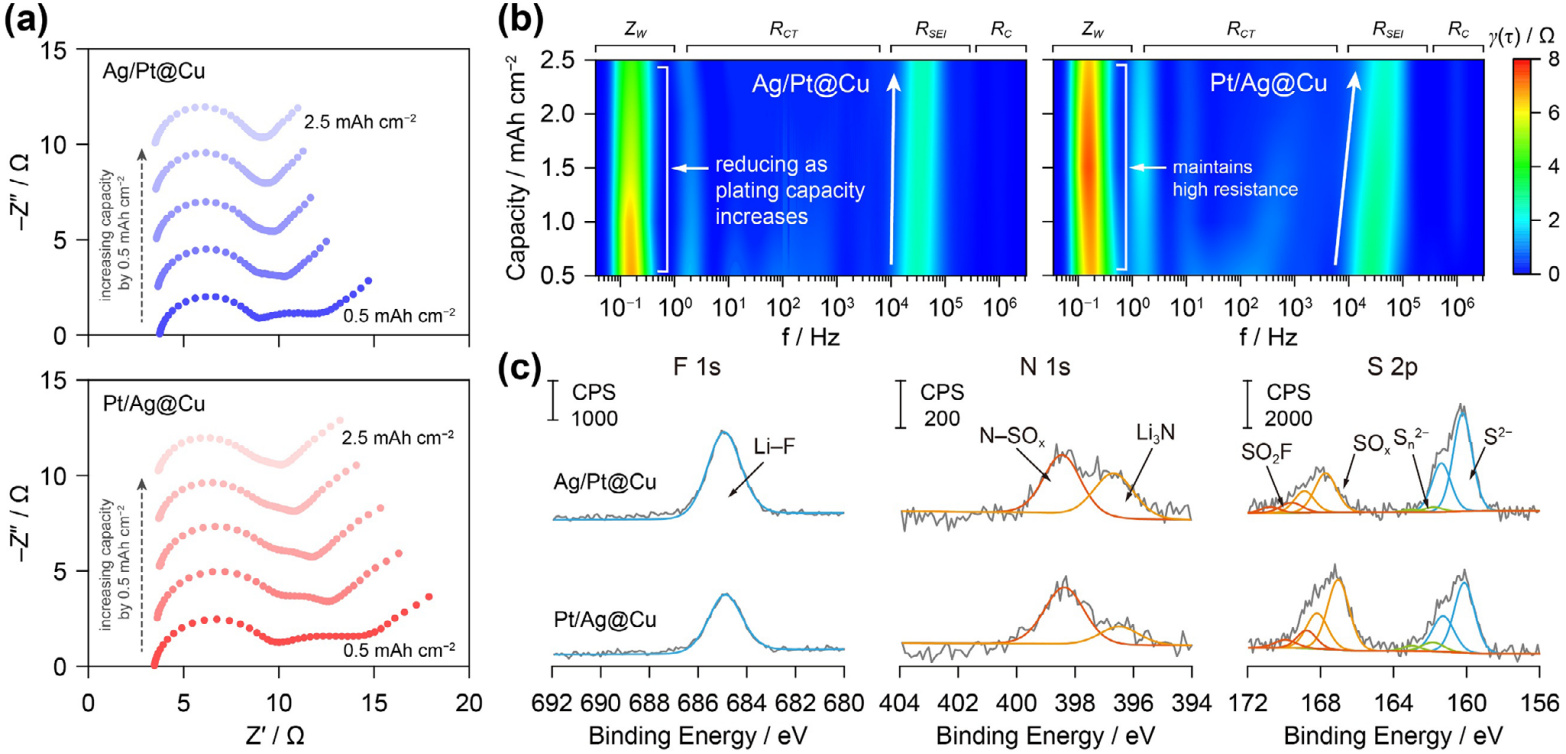
Substrate‐dependent interfacial stability of bilayer coatings. (a) Nyquist plots of Ag/Pt@Cu and Pt/Ag@Cu cells obtained from in situ electrochemical impedance spectroscopy (EIS) during Li plating (0.5–2.5 mAh cm^−2^). (b) 2D intensity color maps of calculated DRT curves corresponding to the EIS results of Ag/Pt@Cu and Pt/Ag@Cu cells. (c) High‐resolution X‐ray photoelectron spectroscopy (XPS) spectra of fluorine (F 1s), nitrogen (N 1s), and sulfur (S 2p) for Ag/Pt@Cu and Pt/Ag@Cu after Li plating to a capacity of 1.0 mAh cm^−2^.

In situ EIS analysis was performed to examine the interfacial evolution of bare Cu, Ag/Pt@Cu, and Pt/Ag@Cu during Li deposition. As shown in Nyquist plots (Figure 4a), the overall resistance decreased in all cases as Li plating progressed, likely due to an enlarged effective surface area originating from progressive Li coverage [50, 51, 52, 53, 54, 55]. Bare Cu seemingly exhibited the lowest resistance, which can be ascribed to non‐uniform Li nucleation that enlarged the surface area (Figure 3a). Pt/Ag@Cu showed the highest resistance at the initial stage, likely due to unstable SEI buildup, followed by the steepest decrease as Li plating continued. In contrast, Ag/Pt@Cu exhibited lower resistance and a more moderate decrease during Li plating, implying more favorable SEI formation and well‐regulated interfacial dynamics. These distinctions are further clarified by the 2D intensity color maps derived from multiple DRT curves (Figure 4b). In the high‐frequency region (10^4^–10^5^ Hz), corresponding to *R*_SEI_ [56, 57], bare Cu (Figure S9) and Ag/Pt@Cu remained relatively consistent, whereas Pt/Ag@Cu exhibited a pronounced peak shift during Li plating, indicative of interfacial instability. In the low‐frequency region (10^0^–10^−1^ Hz), associated with Warburg impedance (*Z*_w_), Pt/Ag@Cu consistently exhibited higher peak intensity compared to bare Cu and Ag/Pt@Cu. This difference is likely attributed to irregular Pt delamination, as confirmed in Figure 3c. Detached Pt domains become embedded within the Li deposit layer, hindering Li diffusion throughout the plating process. Collectively, Ag/Pt@Cu effectively stabilizes the electrolyte‐derived SEI structure, whereas the reverse configuration exacerbates interfacial instability due to Pt‐induced disruption of Li transport.

XPS analysis was performed to examine the chemical nature of the as‐formed SEI on different current collectors after the initial Li plating (1 mAh cm^−2^) (Figure 4c). Since anion‐derived SEI formation in LHCEs is predominantly governed by the unique solvation structure, we primarily examined the F 1s, N 1s, and S 2p spectra to compare the extent of anion decomposition. In F 1s spectra, the strong Li─F peak (∼684.8 eV) was observed at Ag/Pt@Cu, whereas Pt/Ag@Cu showed markedly weaker intensity. The N 1s spectra revealed that Ag/Pt@Cu exhibited pronounced peaks at ∼398.5 eV (N─SO_x_) and ∼396.8 eV (Li_3_N), whereas the lower peak intensity observed for Pt/Ag@Cu may indicate incomplete anion decomposition. Distinct differences were also observed in the S 2p spectra. Bare Cu displayed doublet peaks at 168–170 eV and 161–163 eV, attributed to sulfoxides (SO_x_) and sulfide (S_n_^2−^), respectively. Similarly, Ag/Pt@Cu showed peaks corresponding to the sulfonyl fluoride group (SO_2_F) along with detectable S_n_^2−^, indicating enhanced anion reduction, consistent with the stronger Li─F peak intensity. In contrast, Pt/Ag@Cu exhibited weaker S 2p signals dominated by SO_x_ species, further confirming limited anion‐derived SEI formation at the Pt‐exposed interface. Collectively, direct exposure of the Ag outer surface (Ag/Pt@Cu) facilitates anion adsorption and the formation of anion‐derived SEI, consistent with bare Cu (Figure S10), whereas Pt exposure (Pt/Ag@Cu) impairs these reactions. In addition, Pt debris on the Li deposits may disrupt SEI formation and exacerbate interfacial heterogeneity. To further elucidate the specific benefit of the Ag outer layer, we compared the XPS spectra of Ag/Pt@Cu with those of a single‐layer Pt coating (Pt@Cu). As shown in Figure S11, Ag/Pt@Cu exhibited significantly higher peak intensities for F 1s, N 1s, and S 2p species compared to Pt@Cu. This observation confirms that the Ag outer layer actively facilitates the decomposition of FSI^−^ anions to form a robust, anion‐derived SEI, whereas direct Pt exposure is less effective at promoting these critical interfacial reactions. These experimental findings align with our computational results, highlighting the decisive role of bilayer stacking sequence in governing SEI chemistry and ensuring long‐term interfacial stability.

**FIGURE 5:**
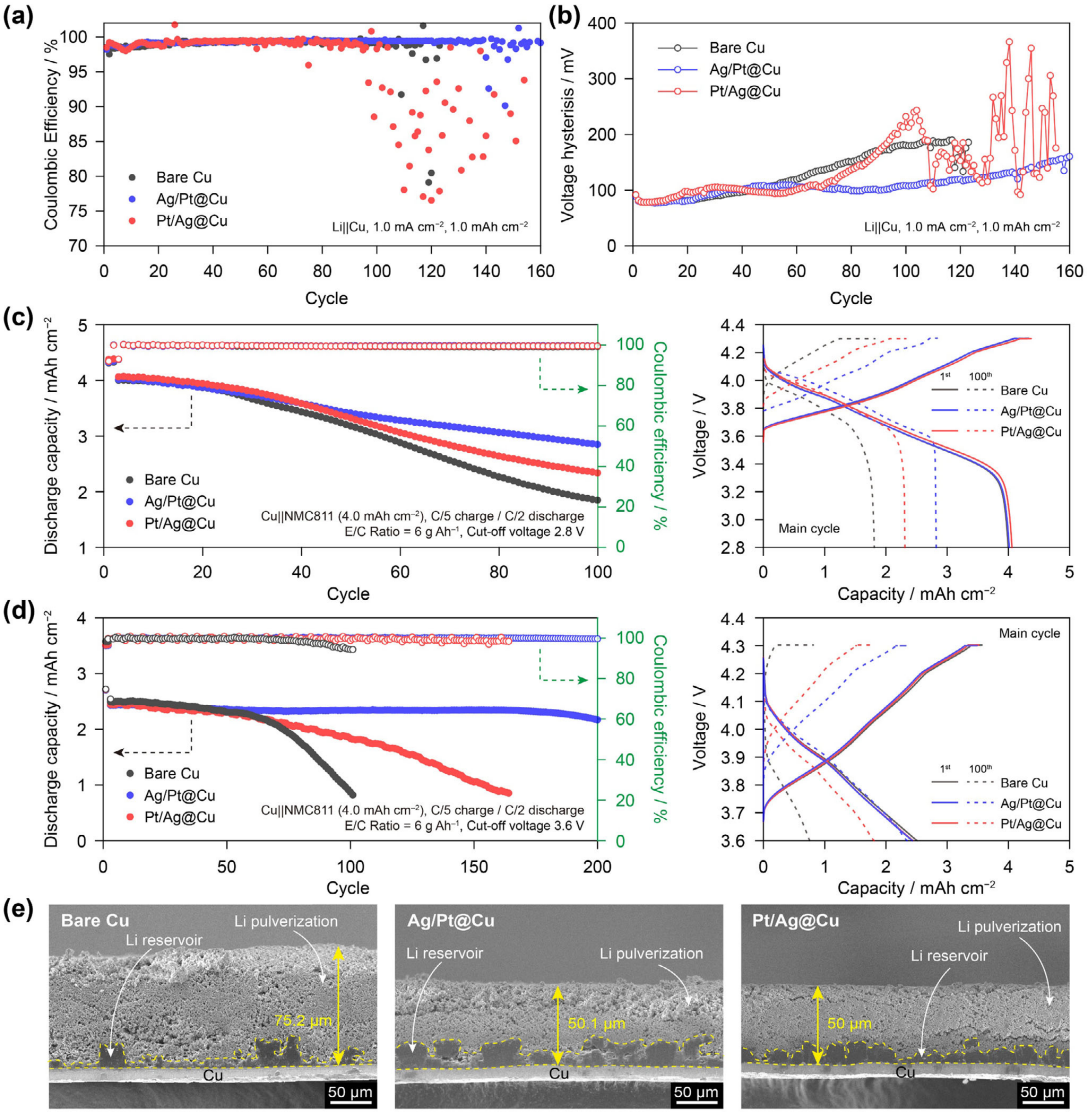
Electrochemical impacts of bilayer coatings on repeated Li plating/stripping. (a) Coulombic efficiency (CE) and (b) voltage hysteresis of Li||Cu cells employing bare Cu, Ag/Pt@Cu, and Pt/Ag@Cu electrodes under full Li utilization at 1.0 mA cm^−2^ with a plating capacity of 1.0 mAh cm^−2^. (c, d) Cycling stability of Cu||NMC811 full cells (4.0 mAh cm^−2^) cycled at C/5 charge and C/2 discharge rates (1C = 4.0 mA cm^−2^), with the state of charge (SOC) controlled to 2.8–4.3 V (c, full utilization) or 3.6–4.3 V (d, partial utilization) versus Li/Li^+^. (e) Cross‐sectional SEM images of the anodes obtained after the 100^th^ discharge step (to 3.6 V cut‐off) under the partial utilization protocol shown in (d).

To amplify the impacts of interfacial stability on reversibility, Li||Cu half cells were cycled using a full Li utilization protocol (Figure 5a). The cell employing bare Cu failed after 120 cycles, showing an abrupt Li CE drop, which is likely attributed to surface deactivation by detrimental Li pulverization [5, 58, 59], as confirmed in progressive increase of voltage hysteresis (Figure 5b). Despite the less dendritic Li deposition in Pt/Ag@Cu, the cell suffered from earlier failure, showing a stiff rise in overpotential and highly unstable voltage profiles beyond 100^th^ cycle (Figure S12). In stark contrast, Ag/Pt@Cu exhibited the most stable retention of Li CEs (∼99.4%) and the smallest increase in plating‐stripping overpotential, leading to minimal voltage hysteresis throughout extended cycling. Thus, lithiophilic coating without interfacial penalty is crucial to sustaining its original structure and securing stable SEI buildup, thereby ensuring long‐term reversibility.

Cu||NMC811 full cell testing further validates the beneficial role of Ag/Pt@Cu in enhancing cycling stability. Under full utilization protocol (Figure 5c), all Li extracted from the cathode (SOC 100%) is repeatedly plated and stripped without leaving residual Li, inevitably exposing the current collector surface and subjecting it to severe structural stress. While the voltage profiles of all cells were nearly identical in the first cycle, the Ag/Pt@Cu cell exhibited superior capacity retention (71% for 100 cycles) and moderate polarization after 100 cycles. In sharp contrast, the bare Cu cell suffered from severe capacity fading (46% retention for 100 cycles) due to uncontrolled interfacial degradation. The Pt/Ag@Cu cell showed somewhat improved capacity retention (57.5%) compared to bare Cu, yet the enhancement remained limited, likely arising from its interfacial instability. Such interfacial instability became even more pronounced in the monometallic Ag@Cu coating (Figure S13), where excessive Ag─Li intermixing accelerated structural degradation.

Under the partial utilization protocol, where 20% of Li was deliberately maintained as a reservoir (Figure 5d), the performance gap became more pronounced, even though residual Li was available to compensate for interfacial degradation. The Ag/Pt@Cu cell demonstrated excellent cycling stability, maintaining 88% capacity retention over 200 cycles, whereas bare Cu and Pt/Ag@Cu cells showed severe capacity fading, only retaining 80% capacity after 70 and 80 cycles, respectively. Given that minimizing cell polarization is a prerequisite for longer cycling under this condition, the combined effects of less dendritic Li plating, structural robustness of the coating layers and stable SEI formation play a decisive role in suppressing polarization. To further distinguish the specific long‐term benefit of the Ag outer layer over the Pt@Cu, we also performed post‐mortem analysis at an earlier stage (30 cycles). Ag/Pt@Cu maintained a significantly thinner residual Li reservoir compared to Pt@Cu, confirming that Ag‐induced stable SEI effectively suppresses Li pulverization from the early stages of cycling (Figure S14). This structural advantage persists throughout extended cycling. Post‐mortem analysis after the 100^th^ discharge step (3.6 V cut‐off) revealed a reacted Li layer thickness of ∼75 µm for bare Cu, but only ∼50 µm for Ag/Pt@Cu and Pt/Ag@Cu, indicating a ∼30% reduction due to suppressed Li pulverization (Figure 5e). Bare Cu, with uncontrolled Li growth, suffered from depletion of residual Li and highly uneven Li utilization across the electrode. Importantly, a substantial amount of available Li was retained in Ag/Pt@Cu, attributable to its enhanced interfacial stability, while Pt/Ag@Cu experienced greater Li depletion due to incomplete SEI formation. When comparing this long‐term observation with the anode state after the first discharge (Figure S15), it becomes evident that the disparity in Li inventory management is established from the very beginning of cycling. Specifically, the thickness of the residual Li reservoir in Ag/Pt@Cu (28.8 µm) was already significantly thinner and more compact than in bare Cu (41.4 µm) and Pt/Ag@Cu (38.8 µm) after the first cycle. This confirms that the Ag outer layer's ability to regulate Li morphology at the onset of cycling is a prerequisite for sustaining a healthy Li inventory, which ultimately dictates the overall cycle life of ZE‐LMBs. The more active Li inventory preserved in Ag/Pt@Cu can buffer interfacial degradation, thereby accounting for its improved cycling stability under SOC‐controlled cycling. The exceptional cycling stability of Ag/Pt@Cu is further highlighted in Table S3, which provides a comprehensive comparison with other reported Cu current collector modification strategies for ZE‐LMBs. Unlike other studies that rely on high external pressure (up to 1,200 kPa) to confine Li growth, our cell achieved superior performance under the relatively low intrinsic pressure of a standard coin cell (∼400 kPa), quantified via pressure‐sensitive film (Figure S16) [60]. Furthermore, while many reported systems operate under ‘flooded’ electrolyte conditions (Table S3), we maintained rigorous lean electrolyte environment with an E/C ratio of 6 g Ah^−1^. The fact that Ag/Pt@Cu maintains superior stability under such lean electrolyte and low‐pressure conditions confirms the robustness of our current collector design.

**FIGURE 6:**
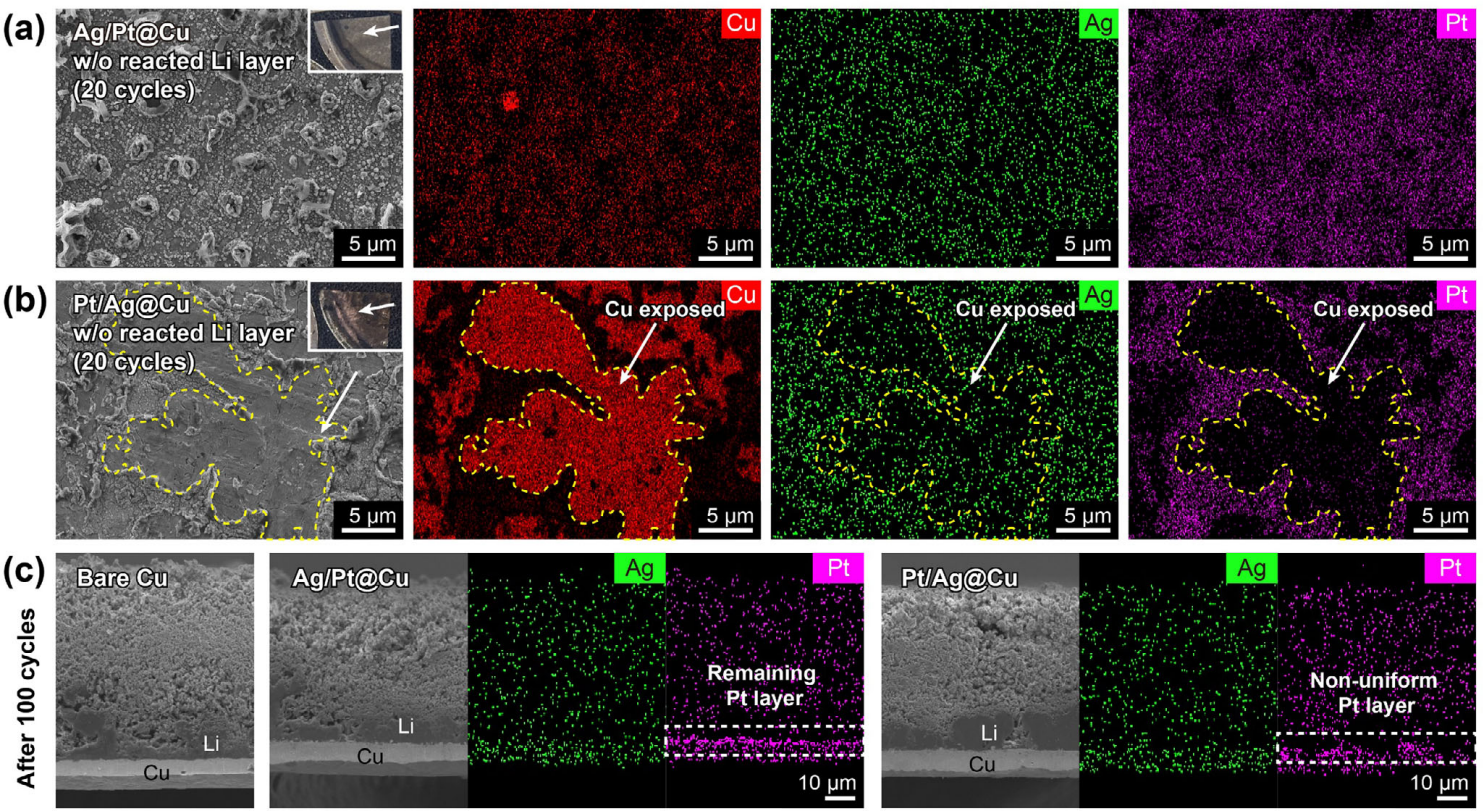
Structural integrity and spatial distribution of the bilayer coatings after electrochemical cycling. Top‐view SEM images and corresponding EDS mappings of (a) Ag/Pt@Cu and (b) Pt/Ag@Cu current collectors. To evaluate the persistence of the coating layers on the substrate, the reacted Li layer was removed after complete Li stripping following 20 cycles of SOC‐controlled partial utilization. (c) Cross‐sectional SEM images and EDS mappings after 100 cycles, illustrating the long‐term spatial evolution of Ag and Pt. This highlights the preserved Pt layer in Ag/Pt@Cu versus the non‐uniform Pt layer in Pt/Ag@Cu.

To unveil the structural stability and distribution of bilayer metallic coatings during and post‐cycling, we conducted post‐mortem SEM and EDS analyses (Figure 6). After 20 cycles, residual Li was completely stripped, and the reacted Li layer was removed without mechanical damage to the current collectors, to assess the preservation of the original coating layer. The reacted Li layer was easily removed from bare Cu, where no residual SEI or “dead” Li was detected (Figure S17). Unlike bare Cu, the Ag/Pt@Cu surface exhibited a roughened morphology with clear Li plating imprints. EDS mapping revealed the presence of Pt across the Ag/Pt@Cu surface (Figure 6a), except for occasional Cu exposure likely caused by partial detachment of the coating during sample preparation. This suggests that the Ag/Pt@Cu configuration can preserve its original bilayer structure even after repeated Li plating and stripping. In contrast, most of the coating layers on Pt/Ag@Cu disappeared, non‐uniformly exposing the underlying Cu and producing a more heterogeneous surface (Figure 6b). EDS mapping further revealed strong Cu signals in local areas where neither Pt nor Ag was detected, confirming the severe loss of both metal layers during Li pulverization. Post‐mortem analyses were further conducted on samples subjected to SOC‐controlled cycling for 100 cycles to evaluate the spatial evolution of both metals. Cross‐sectional EDS mapping directly revealed that while Ag was distributed throughout the porous reacted layer in both configurations, the Pt distribution showed a pronounced dependence on the coating sequence. Pt/Ag@Cu retained only discontinuous, sparsely distributed Pt clusters, whereas Ag/Pt@Cu preserved a continuous Pt band adherent to the Cu current collector even after 100 cycles (Figure 6c). Beyond simply suppressing dendrites at the initial stage, maintaining structural stability in lithiophilic coating layers without incurring interfacial penalty is crucial for ensuring the cycling stability of ZE‐LMBs.

## 3 Conclusion

In this study, we demonstrated a bimetallic bilayer coating strategy to address the limitations of single‐layer lithiophilic coatings on current collectors in ZE‐LMBs. Through DFT analyses and electrochemical verifications, we established the design principles by exploiting two Li‐alloyable metals, Ag and Pt: Ag as the outer layer promotes uniform Li nucleation and robust anion‐derived SEI formation owing to moderate Li binding and strong anion adsorption, whereas Pt as the inner layer acts as structural and interfacial support, sustaining long‐term lithiophilicity. Through functional decoupling in a bilayer design, Ag/Pt@Cu enables conformal Li deposition, stable SEI formation, and structural integrity even under dynamic interfacial conditions. Positioning Ag as the outer layer also buffers the structural fragility of Pt during Li alloying, thereby preserving long‐term lithiophilicity. In the reverse structure, Pt/Ag@Cu suffered from interfacial instability and structural degradation caused by volume mismatch and Pt delamination, ultimately leading to structural collapse and poor cycling. This, in turn, suggests that optimizing surface lithiophilicity alone cannot guarantee improved cycling performance; reinforcing structural stability must be rationally engineered by carefully selecting coating metals and optimizing their sequence and composition. Beyond simple dendrite suppression, this planar bilayer configuration provides a practical and energy‐dense design framework that effectively overcomes the volumetric and processing complexities typical of 3D‐structured or composite anodes, while enhancing the persistence of lithiophilicity for longer and more stable cycling of ZE‐LMBs.

## Conflicts of Interest

The authors declare no conflicts of interest.

## Supporting information




**Supporting File**: advs75045‐sup‐0001‐SuppMat.docx.

## Data Availability

The data that support the findings of this study are available from the corresponding author upon reasonable request.
