# Supplementary material for: Lithiophilic Current Collector Without Interfacial Penalty in Zero‐Excess Lithium Metal Batteries
Source: Adv Sci (Weinh). 2026 Mar 28;13(33):e19303. doi: 10.1002/advs.202519303 (PMC13271633; doi:10.1002/advs.202519303)
Supplement: Supplementary file 1 — Supporting File: advs75045‐sup‐0001‐SuppMat.docx. [file ADVS-13-e19303-s001.docx]

Supporting Information

**Lithiophilic Current Collector without Interfacial Penalty in Zero-Excess Lithium Metal Batteries**

*Jiyeon Seo*^1,2,†^, *Sangseob Lee*^1,†^, *Seojin Jeon*^3,†^, *Minhong Lim*^1^, *Hyegang Koo*^3^, *Seung-Tae Hong*^2^, *Woosun Jang*^3,4,^*, *Aloysius Soon*^1,^*, *Hongkyung Lee*^1,2,3,^*

^1^Department of Materials Science and Engineering, Yonsei University, Seoul 03722, Republic of Korea

^2^Department of Energy Science and Engineering, Daegu Gyeongbuk Institute of Science and Technology (DGIST), Daegu 42988, Republic of Korea

^3^Department of Battery Engineering, Yonsei University, Seoul 03722, Republic of Korea

^4^Integrated Science and Engineering Division, Yonsei University, Incheon 21983, Republic of Korea

*Emails: [woosunjang@yonsei.ac.kr](mailto:woosunjang@yonsei.ac.kr) (W.J.); [aloysius.soon@yonsei.ac.kr](mailto:aloysius.soon@yonsei.ac.kr) (A.S.); [hongkyung.lee@yonsei.ac.kr](mailto:hongkyung.lee@yonsei.ac.kr) (H.L.)

**Experimental Section**

*Material preparations*: Commercial Cu foil (10 μm thick) was used as the base current collector substrate (denoted as bare Cu). Ultrathin metal coatings were deposited onto the Cu substrates using a DC-sputtering instrument (CCU-010 HV, Safematic, Switzerland). For bilayer configurations, Ag and Pt were sequentially deposited. Two bilayer structures were fabricated by coating 25 nm Pt bottom layer followed by 25 nm Ag top layer for Ag/Pt@Cu and vice versa for Pt/Ag@Cu. A localized high-concentration electrolyte (LHCE), adopted from previous studies,^[1]^ was prepared by dissolving lithium bis(fluorosulfonyl)imide (LiFSI) in 1,2-dimethoxyethane (DME) and 1,1,2,2-tetrafluoroethyl-2,2,3,3-tetrafluoropropyl ether (TTE) with a molar ratio of 1:1.2:3. A polyethylene (PE) separator (19 mm diameter) was also used for all the coin cell assemblies.

*Electrochemical measurements*: All cells investigated in this work were assembled in coin-type cells (CR2032, Welcos) within an Ar-filled glove box, without applying any additional external pressure. For the Li||Cu half-cells, Li metal foil (200 μm thick, 16 mm diameter) was used as both counter and reference electrodes, while bare Cu and M-Cu working electrodes were prepared as 19 mm diameter discs. A fixed volume of 75 μL of electrolyte was injected into each cell. To evaluate Li Coulombic efficiency (CE), Li is deposited at a current density of 1.0 mA cm^−2^ with a fixed areal capacity of 1.0 mAh cm^−2^, followed by stripping up to 1 V. The average CE for a given number of cycles ($n$) was calculated as:

$$\mathrm{CE}_{1}=\frac{1}{n}\sum_{i=1}^{n} \frac{Q_{S,i}}{Q_{T,i}}\times100\% (1)$$

where *Q*_T,_*_i_* is the Li deposition capacity (1.0 mAh cm^−2^), *Q*_S,_*_i_* is the stripping capacity in each cycle.^[2]^ For the Cu||LiNi_0.8_Mn_0.1_Co_0.1_O_2_ (NMC811) full cells, cathodes with the areal capacity of 4.0 mAh cm^−2^ were used. A fixed volume of 32.4 µL of electrolyte was injected in each cell, corresponding to an electrolyte-to-capacity (E/C) ratio of 6 g Ah^−1^. Two formation cycles were performed at 0.1 C rate within the voltage range of 3.6–4.3 V at 25 °C. Subsequent cycling was performed at a C/5 charge (constant current/constant voltage, CC/CV mode) and a C/2 discharge (CC mode). Cells were discharged to either 3.6 V or 2.8 V to examine how the presence of residual Li at the anode influences electrochemical performance.

*Characterizations*: Li plating morphology on bare Cu, Ag/Pt@Cu, and Pt/Ag@Cu was analyzed using a field-emission scanning electron microscope (FE-SEM, JSM-IT800(SHL), JEOL Ltd.) after depositing capacities of 0.1 mAh cm^−2^ and 1.0 mAh cm^−2^ at a current density of 0.5 mA cm^−2^. For post-mortem analysis, all cycled cells were disassembled in an Ar-filled glove box, washed with DME solvent (Enchem, Korea), used in the electrolyte, and dried under ambient vacuum conditions. The chemical components of the SEI on the current collector surfaces were examined using X-ray Photoelectron Spectroscopy (XPS, K-alpha, Thermo U. K.). For peak deconvolution, the Casa XPS program was used, and the C 1s peak (284.8 eV) was used to calibrate the binding energies.

*Computational studies*: All Density Functional Theory (DFT) calculations were performed using the projector augmented wave (PAW) method as implemented in the Vienna Ab initio Simulation Package (VASP).^[3-5]^ Perdew-Burke-Ernzerhof (PBE) type of exchange-correlation functional was throughout this study,^[6]^ while the optB86b-vdW exchange-correlation functional, a self-consistent non-local van der Waals correction, was employed in calculating the anionic adsorption cases to properly mimic the impact of vdW-type weak forces.^[7]^ Geometries were optimized until the forces on all unconstrained atoms were below 0.01 eV Å^-1^ and the total energy difference between self-consistent steps was less than 10^-5^ eV. A plane-wave kinetic energy cutoff of 500 eV (600 eV for Li-containing structures) was used, with a Γ-centered k-point grid corresponding to a spacing between adjacent k-points being 0.15 Å^-1^. The choice of k-point density was validated through a comprehensive energy convergence test for all studied systems, ensuring that the total energy was converged within $0.02 eV atom^−1^ (Figure S1).

For surface calculations, we modelled various surface indices with different surface slab thickness, to ensure its convergence in energies. Bottommost 2 layers (or at the center if symmetric cell is used) were fixed to mimic the bulk region, while the other layers were allowed to relax freely. A vacuum layer larger than 15 Å was ensured along surface-normal direction (z-direction) to prevent unphysical interactions between periodic slabs, with applied dipole correction when using asymmetric slab structure. The surface energy ($\gamma$) was calculated using the following equation,

$\gamma= \frac{1}{2A} (E_{slab}-nE_{bulk})$,

where $E_{slab}$, $E_{bulk}$, $n$, and $A$ is for the total energy of the surface slab, total energy of corresponding bulk structure, number of atoms in a slab, and surface area, respectively. The adsorption energy (*E*^ads^) of the adsorbate on top of the slab was calculated using the following equation:

$E^{\mathrm{ads}}= E_{slab+ads}-E_{\mathrm{slab}}-E_{adsorbate}$,

where $E_{slab+ads}$, $E_{\mathrm{slab}}$, and $E_{adsorbate}$ denoting the energy of the slab with the adsorbate species, the clean slab, and the isolated adsorbates, respectively. The dissociation energy (*E*^diss^) for the F bond was calculated as:

$E^{\mathrm{diss}}= E_{slab+ads-F}-E_{slab+ads}-0.5*E_{F_{2}}$,

where $E_{slab+ads-F}$ and $E_{F_{2}}$ are the energy of the slab after F dissociation, and F_2_ molecule in the gas phase, respectively. To provide a more holistic perspective in SEI formation energetics, the ensemble-averaged energy ($E_{ens,M}$) was obtained by applying Boltzmann weighting to the accessible states under two major adsorbate configurations (*c* = 1N and 3O),

$E_{ens,M}= \sum_{c} (p_{M,c}^{\mathrm{ad}}*E_{M,c}^{\mathrm{ad}})+ \sum_{c} (p_{M,c}^{\mathrm{diss}}*E_{M,c}^{\mathrm{diss}})$ ,

where $p_{M,c}^{\mathrm{ad}}$, $E_{M,c}^{\mathrm{ad}}$, $p_{M,c}^{\mathrm{diss}}$, and $E_{M,c}^{\mathrm{diss}}$ are the Boltzmann probability of configuration *c* on metal M in the adsorption ensemble, the adsorption energy of corresponding state, the Boltzmann probability of dissociation under configuration *c*, and the corresponding dissociation energy, respectively. Here, the Boltzmann probabilities are evaluated as:

$p_{M,c}^{\mathrm{ad}}=exp(\frac{-E_{c}^{\mathrm{ad}}}{k_{b}*T}) /\sum_{s} exp(\frac{-E_{s}^{\mathrm{ad}}}{k_{b}*T})$, and $p_{M,c}^{\mathrm{diss}}=exp(\frac{-E_{c}^{\mathrm{diss}}}{k_{b}*T}) /\sum_{s} exp(\frac{-E_{s}^{\mathrm{diss}}}{k_{b}*T})$,

where $k_{b}$ and *T* are Boltzmann constant and temperature, respectively.


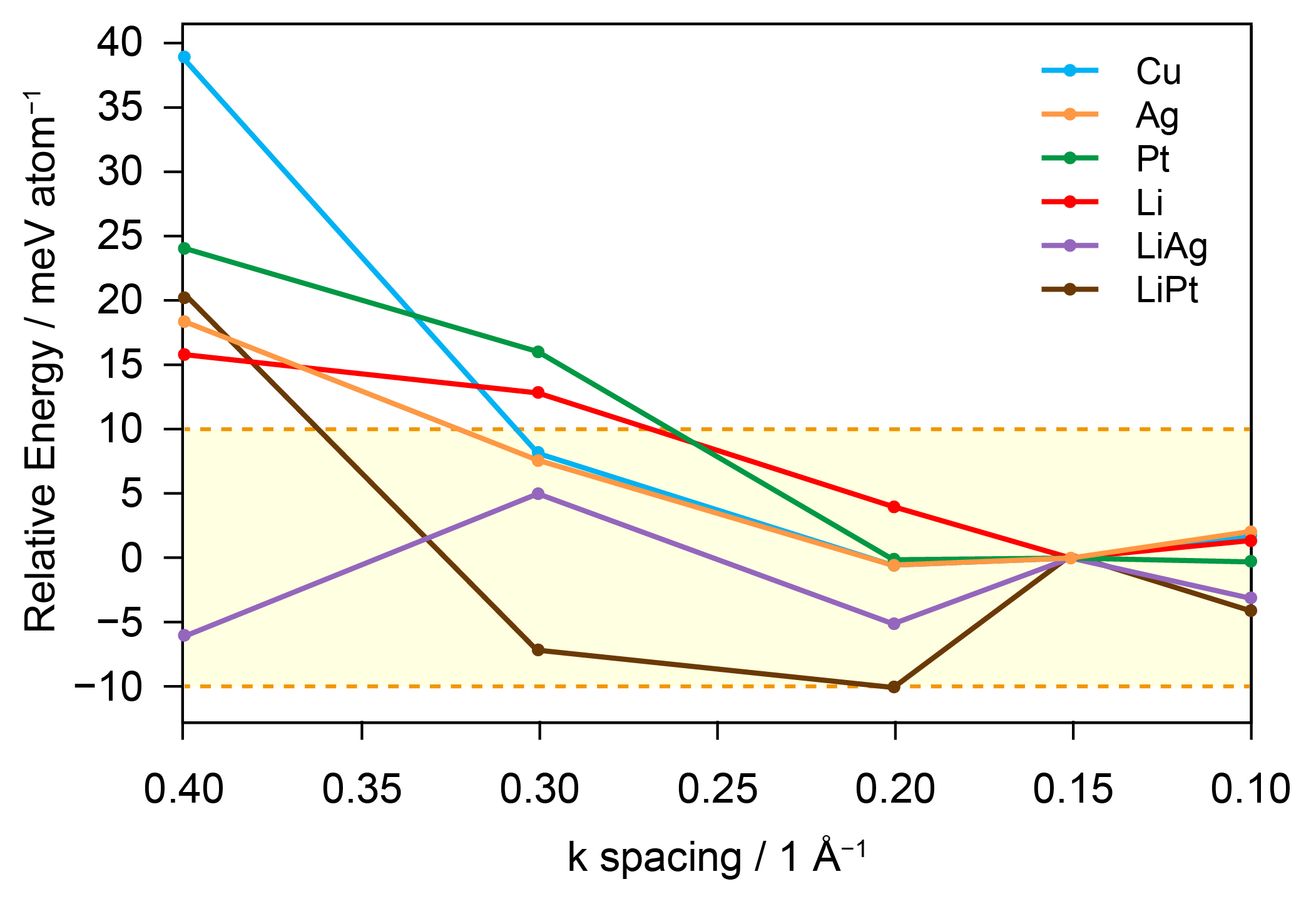


**Figure S1**. k-point convergence test for various systems. The relative energy (eV atom^−1^) for Cu, Ag, Pt, Li, LiAg, and LiPt is plotted as a function of reciprocal k-point spacing (Å^−1^). All energies are referenced to the value at $0.15 Å^−1^(our calculation condition). The yellow shaded region represents an energy window of 0.02 eV atom^−1^ (±10 meV atom^−1^), demonstrating that the chosen k-spacing provides sufficient convergence for all calculated properties.


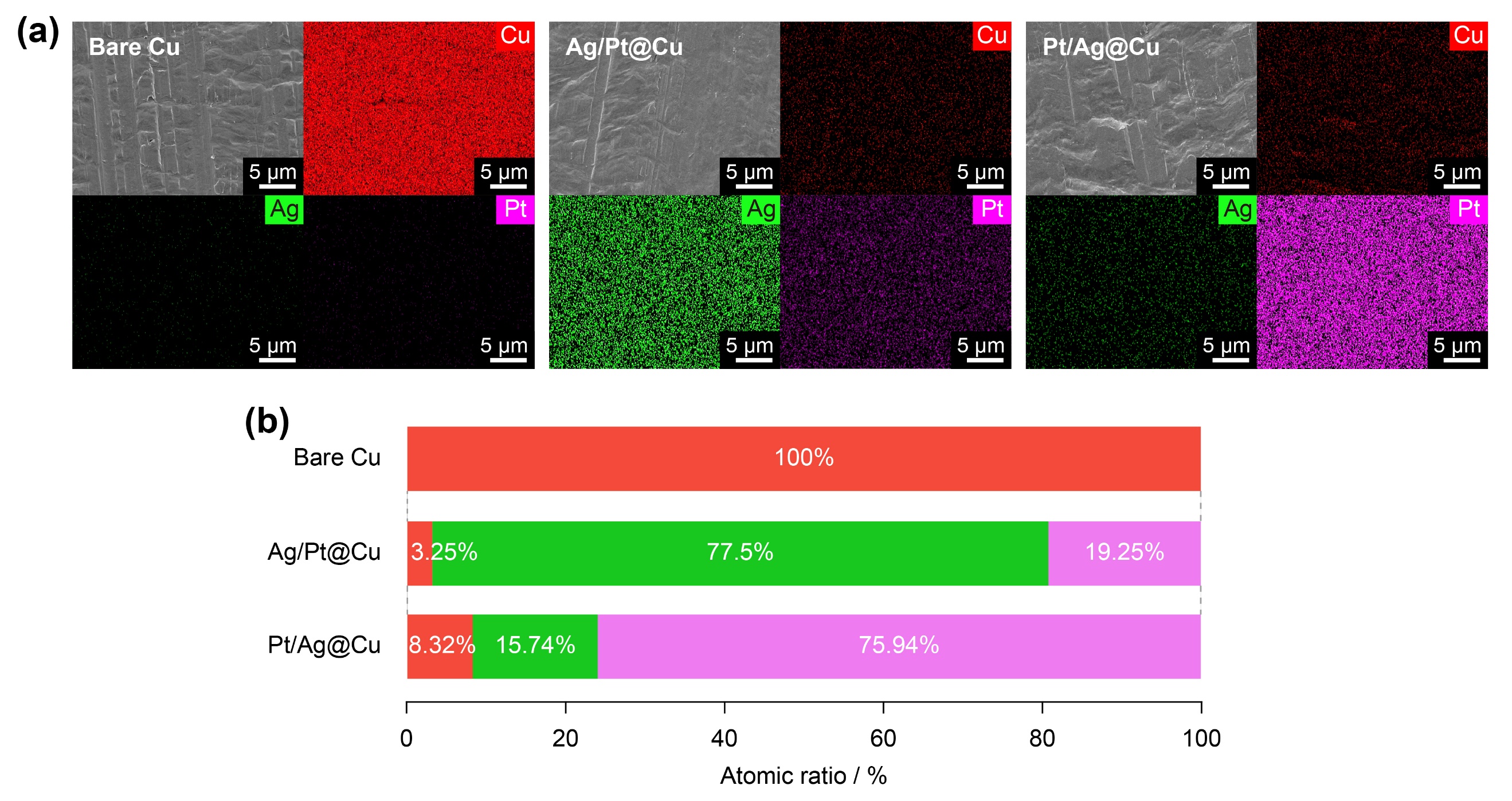


**Figure S2.** (a) Top scanning electron microscopy (SEM) images and corresponding energy-dispersive X-ray spectroscopy (EDS) elemental mappings of bare Cu, Ag/Pt@Cu, and Pt/Ag@Cu electrodes. (b) Atomic compositions (at.%) from EDS quantification.

The Ag/Pt@Cu surface, Ag dominated the surface (77.5 at.%) with a minor Pt contribution (19.3 at.%), confirming Ag as the outer layer. Conversely, Pt/Ag@Cu exhibited Pt as the major surface component (75.9 at.%), consistent with Pt being the top layer. A quantitative comparison further shows that the outer-layer metals account for ≥75 at.% on each bilayer sample, collectively verifying the successful formation of the intended Ag–outer/Pt–inner (Ag/Pt@Cu) and Pt–outer/Ag–inner (Pt/Ag@Cu) coatings.


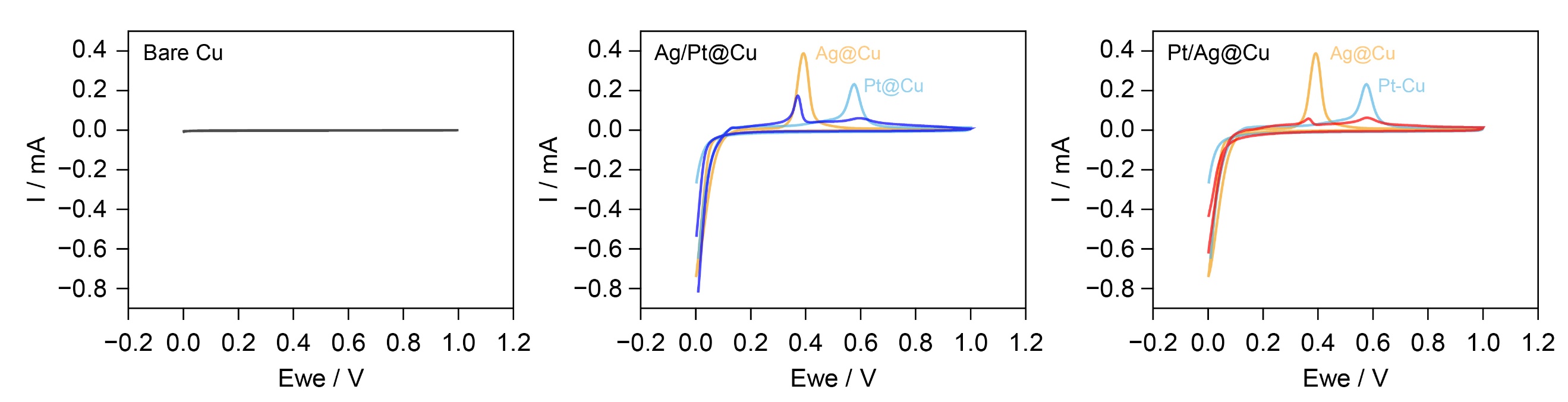


**Figure S3.** Cyclic voltammetry (CV) curves of bare Cu, Ag/Pt@Cu and Pt/Ag@Cu at a scan rate of 0.5 mV s^−1^ within 0.001–1.0 V vs. Li/Li^+^.


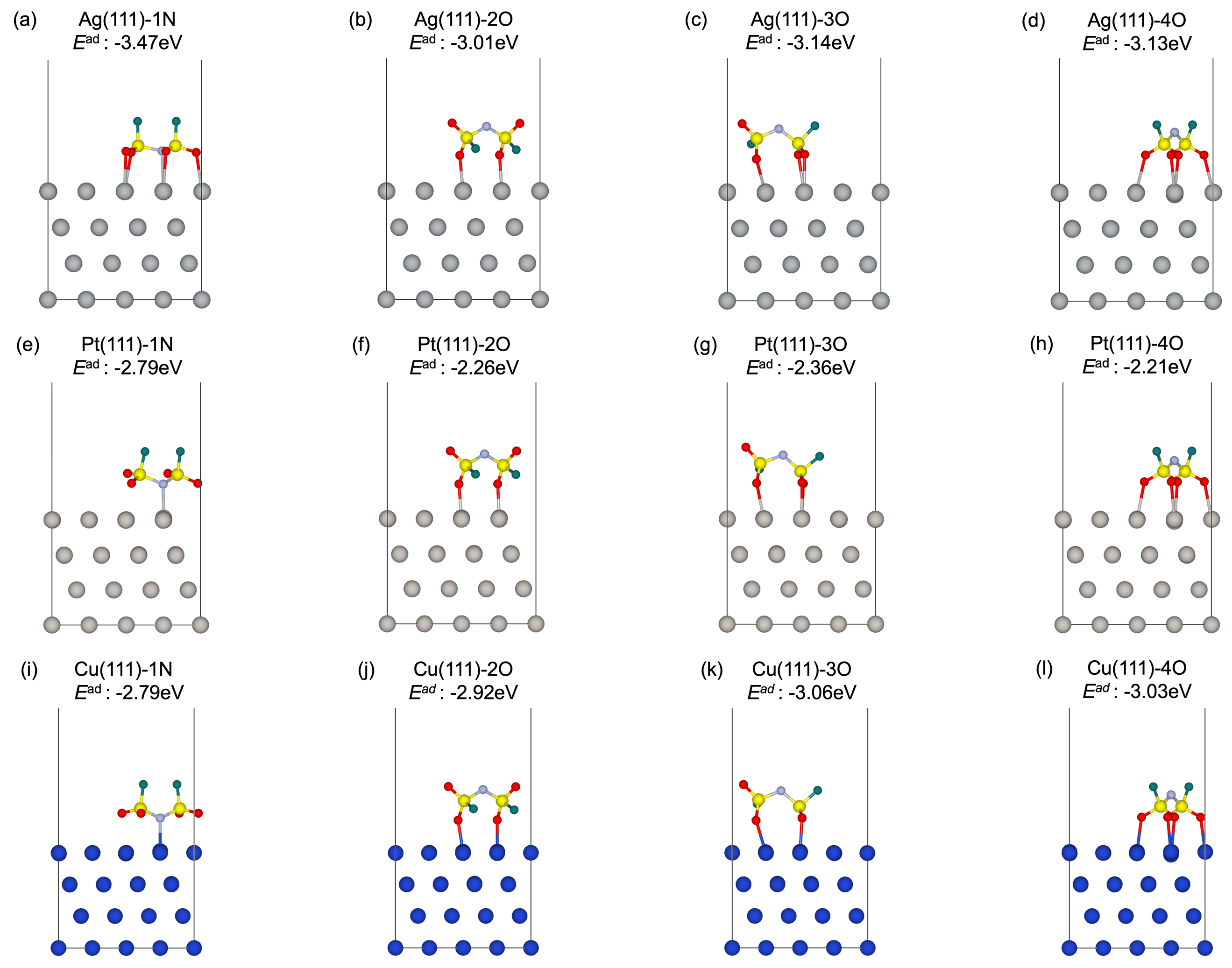


Figure S4. Various adsorption configurations of the N(SO_2_F)_2_ anion on Ag(111), Pt(111), and Cu(111) surfaces. The corresponding adsorption energy (*E*^ad^) is provided in eV above each structure. Ag, Pt, Cu, N, S, O, and F atoms are depicted in gray, beige, blue, sky blue, yellow, red, and green, respectively.


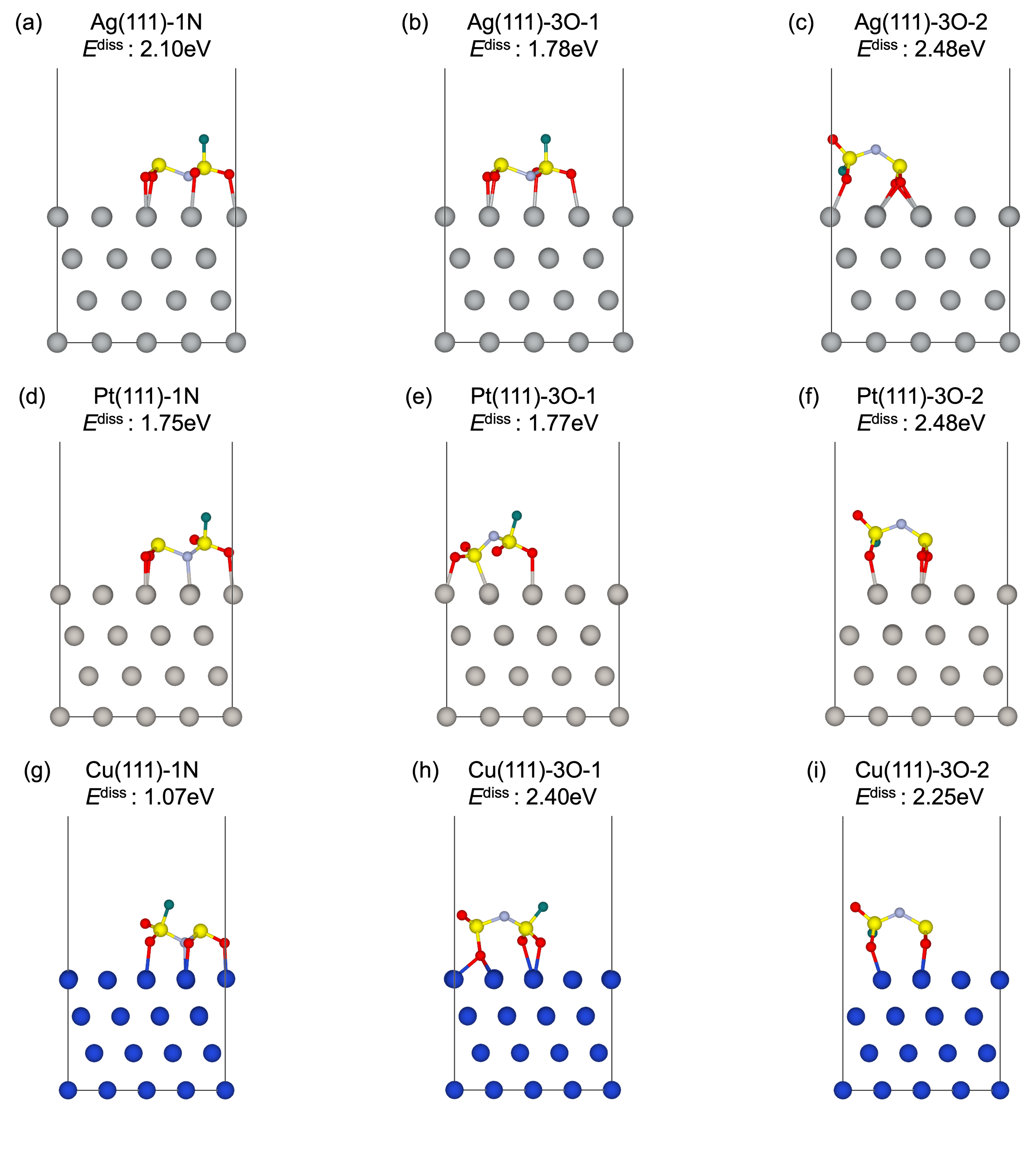


Figure S5. Calculated dissociation energies (*E*^diss^) for a fluorine atom from the N(SO_2_F)_2_ anion on different metal surfaces. The final relaxed atomic configurations are shown for dissociation from the 1N state, and from the two non-equivalent fluorine sites in the 3O state (labeled 3O-1 and 3O-2). Ag, Pt, Cu, N, S, O, and F atoms are depicted in gray, beige, blue, sky blue, yellow, red, and green, respectively.


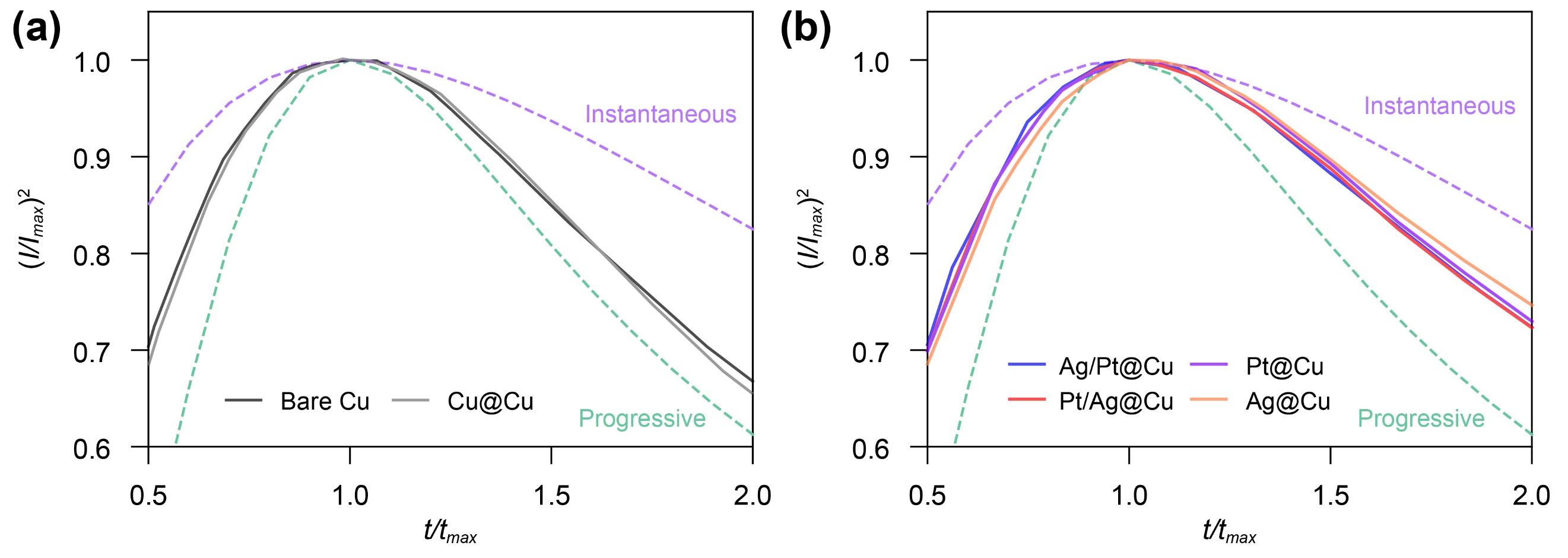


**Figure S6.** Non-dimensional plot of (a) bare Cu, Cu@Cu, and (b) bilayer (Ag/Pt@Cu and Pt/Ag@Cu) and single-layer (Pt@Cu and Ag@Cu) coatings derived from experimental time-current transient curves from chronoamperometry (CA) test, including theoretical curves for instantaneous and progressive nucleation.


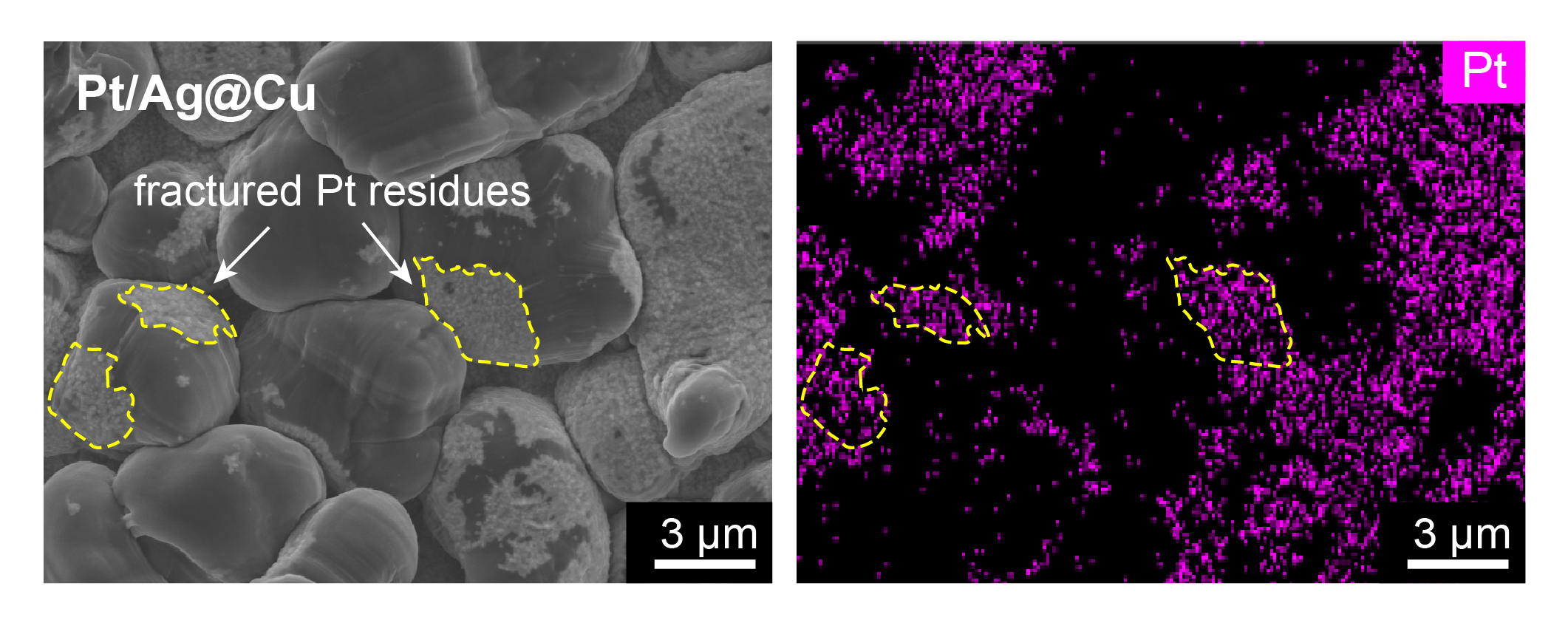


**Figure S7.** Top-view SEM and EDS mappings of Pt/Ag@Cu after Li plating (1.0 mAh cm^−2^) at 0.5 mA cm^−2^.


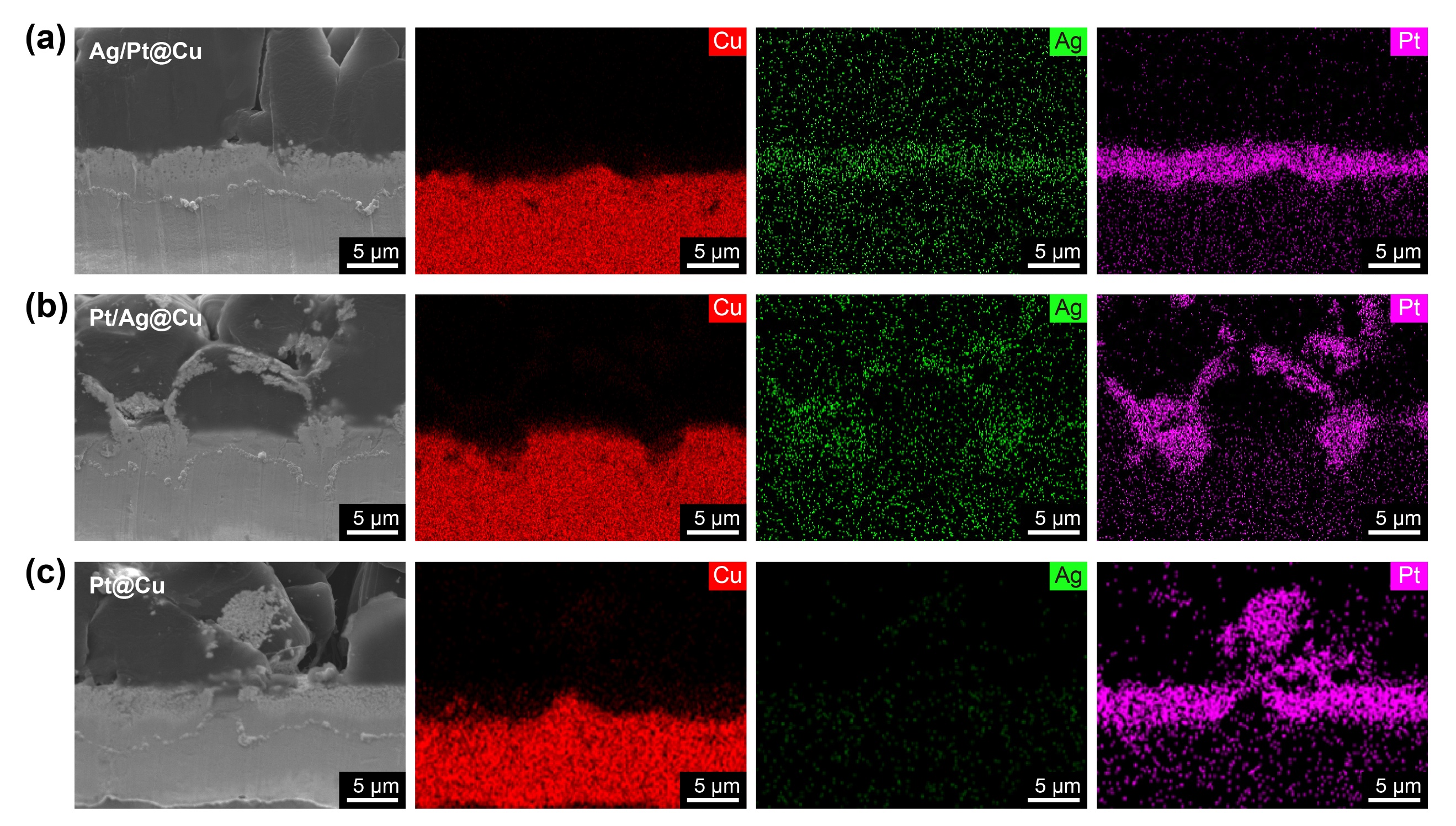


**Figure S8.** Magnified version of cross-section SEM and corresponding EDS mapping for (a) Ag/Pt@Cu, (b) Pt/Ag@Cu and (c) Pt@Cu after Li plating (1.0 mAh cm^−2^) with the current density fixed at 0.5 mA cm^−2^ (magnified version of Figure 3c).


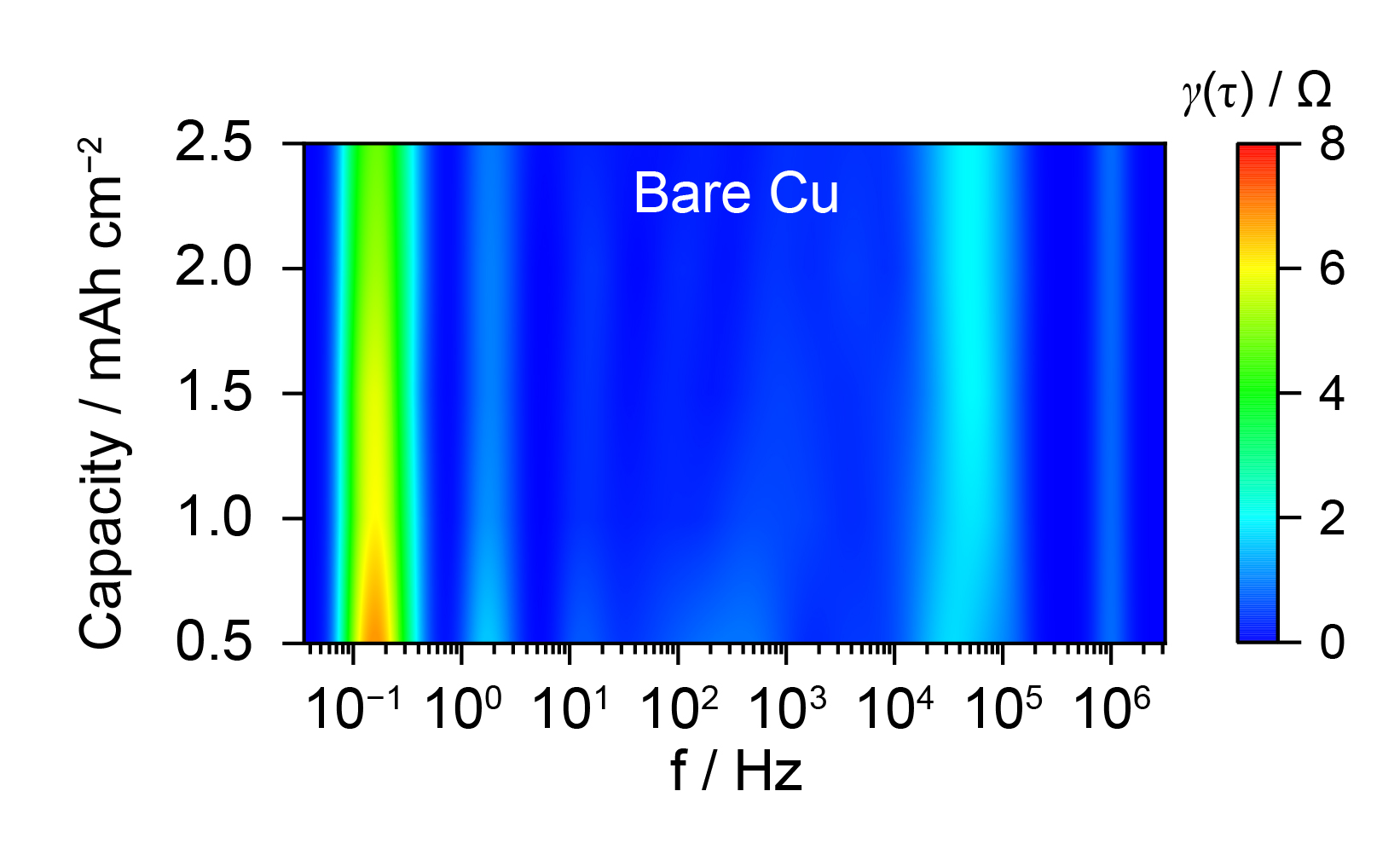


**Figure S9.** 2D intensity color map of calculated DRT curve of bare Cu.


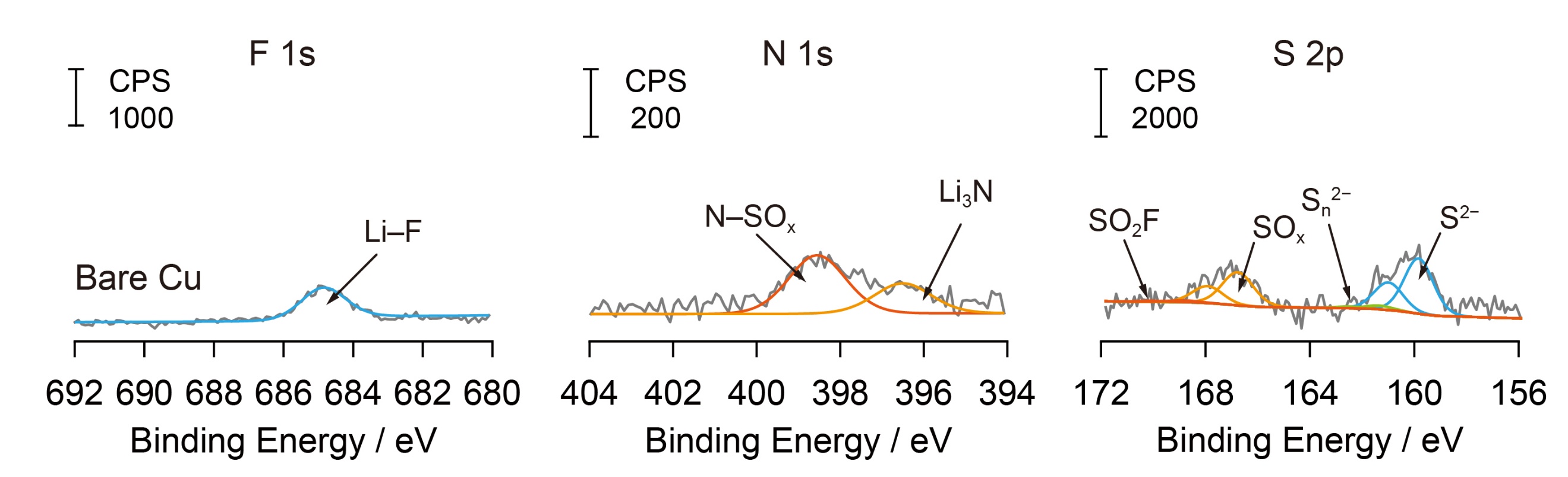


**Figure S10.** High-resolution XPS spectra for N 1s, F 1s, and S 2p presented for bare Cu after Li plating with the capacity of 1.0 mAh cm^−2^.


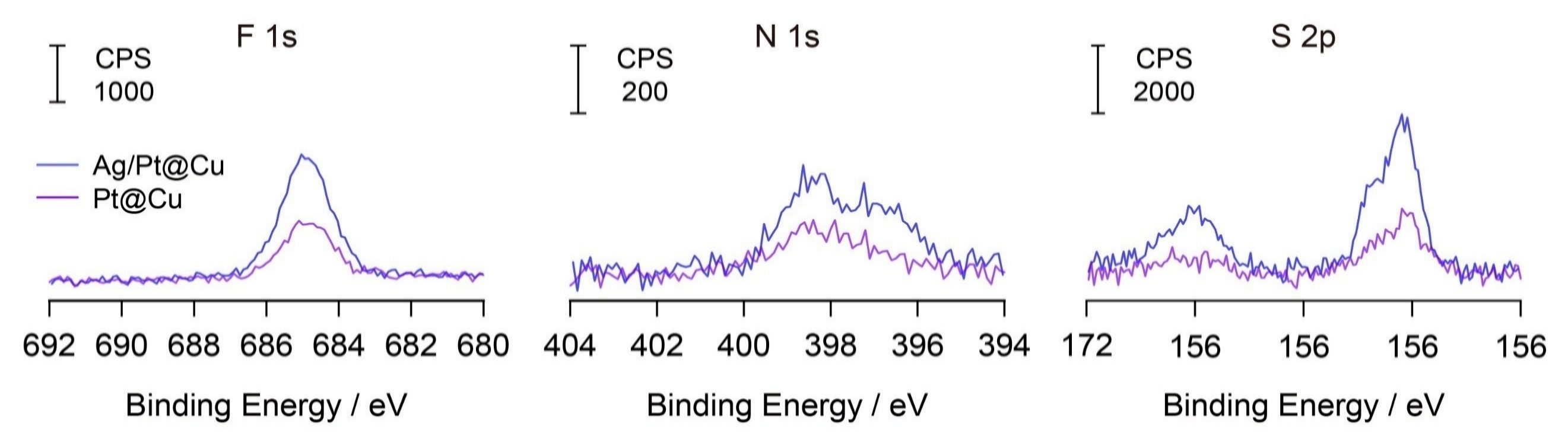


**Figure S11**. Comparison of F 1s, N 1s, and S 2p XPS spectra for Ag/Pt@Cu and Pt@Cu after Li plating (1.0 mAh cm^−2^).


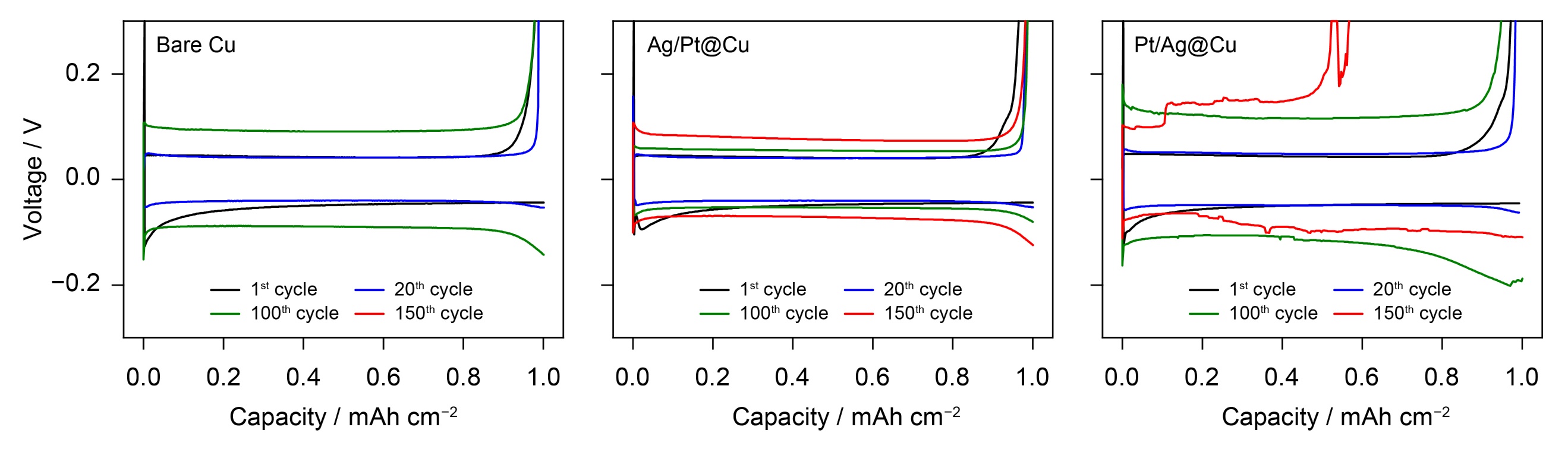


**Figure S12.** Capacity-voltage curve of Li||Cu half cells of each electrode with plating capacity of 1.0 mAh cm^−2^ and stripping up to 1 V to fully expose the current collector, with fixed current density of 1.0 mA cm^−2^.


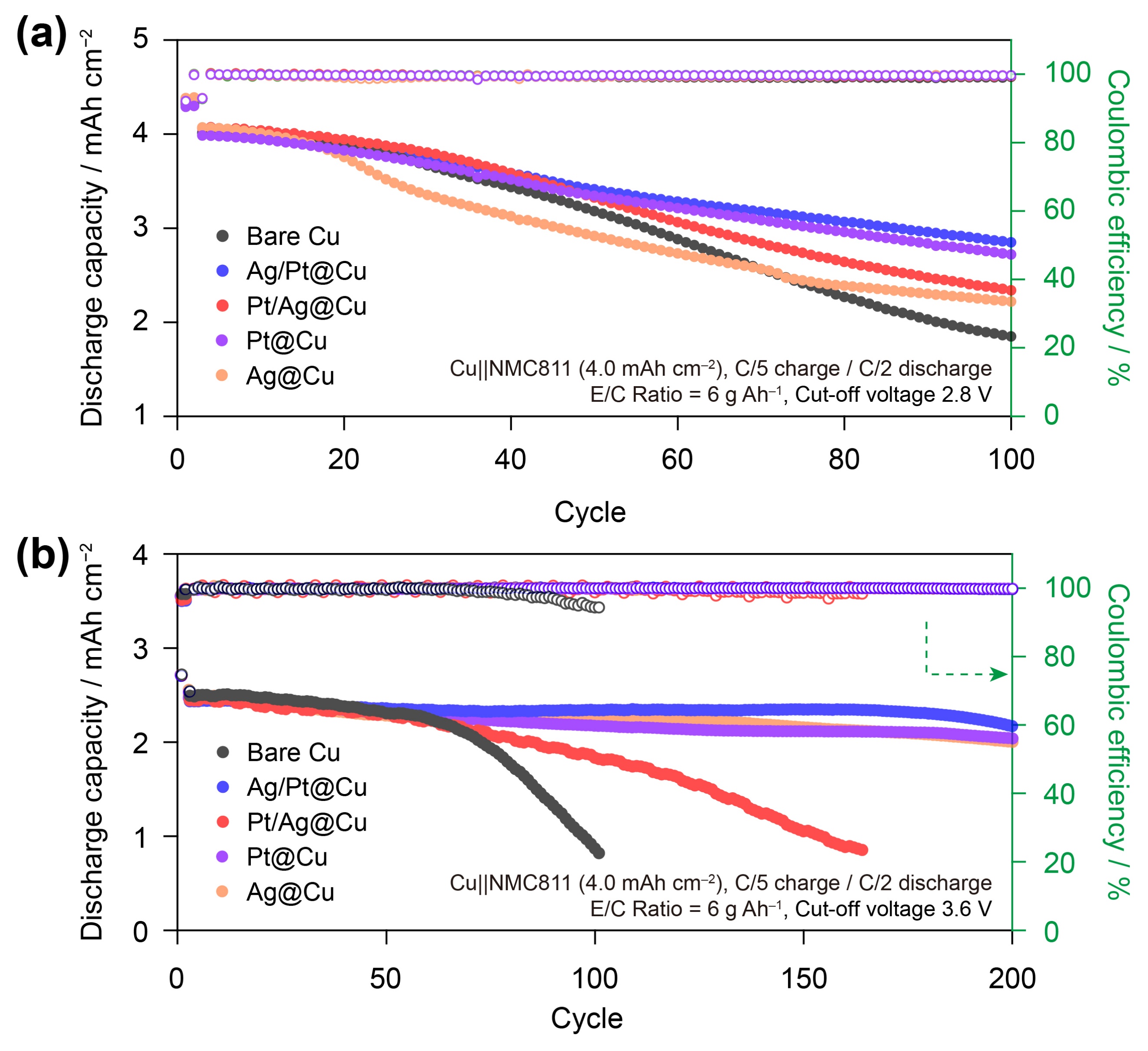


**Figure S13.** Cycling performance of Cu||NMC811 (4.0 mAh cm⁻²) at C/5 charge and C/2 discharge rates (1 C = 4.0 mA cm⁻²). Cu includes bare Cu, bilayer (Pt/Ag@Cu and Ag/Pt@Cu) and single-layer (Pt@Cu and Ag@Cu) coatings.


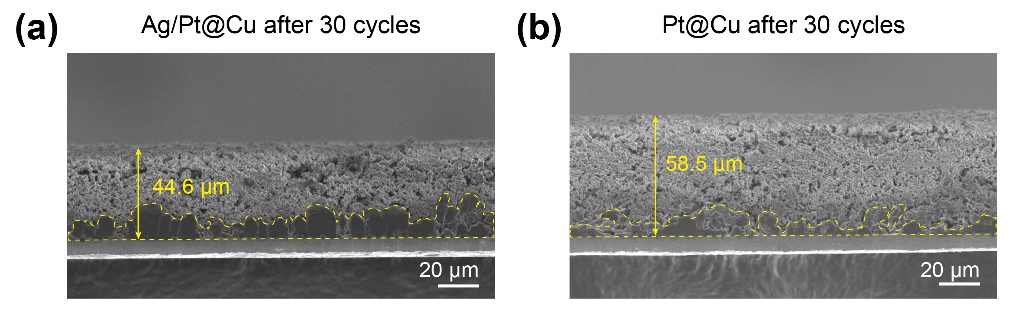


**Figure S14**. Post-mortem cross-sectional SEM images of the anode from the Cu||NMC811 full cells (4.0 mAh cm^−2^) cycled at C/5 charge and C/2 discharge rates (1C = 4.0 mA cm^−2^), with the state of charge (SOC) controlled to 3.6–4.3 V (d, partial utilization) versus Li/Li^+^ after 30 cycles, comparing (a) Ag/Pt@Cu and (b) Pt@Cu current collectors.


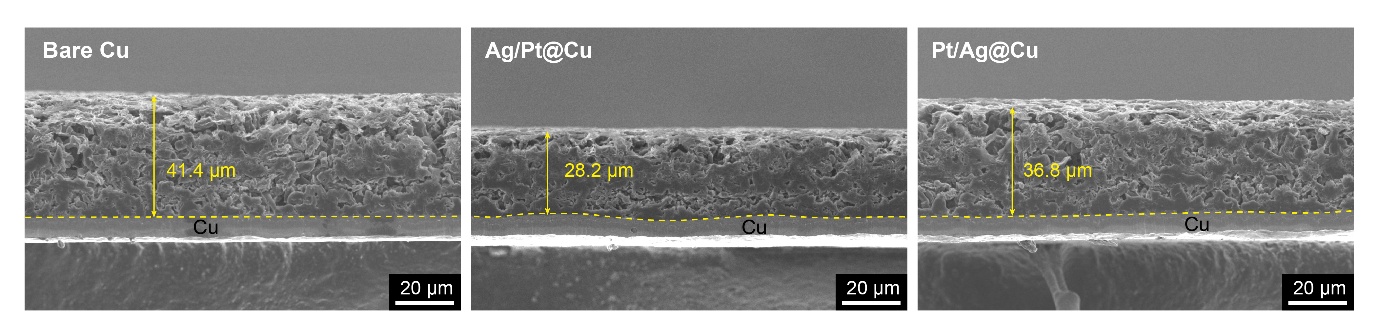


**Figure S15.** Cross-sectional SEM images of the anodes obtained after main 1^st^ cycle discharge step under the cycling condition in partial utilization (3.6–4.3 V).


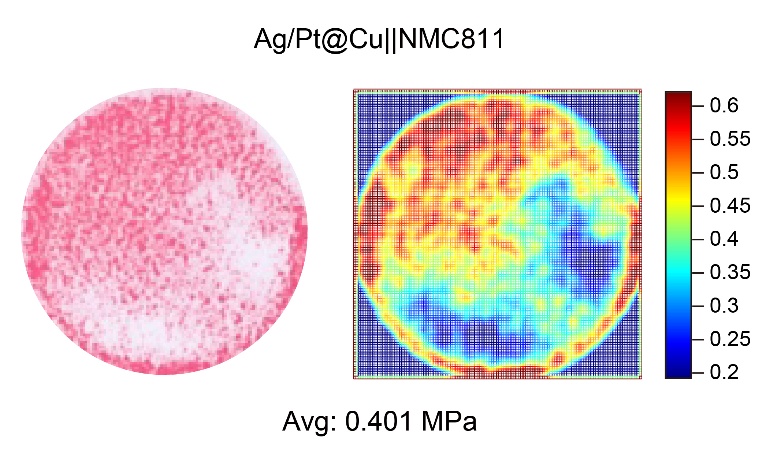


**Figure S16.** Quantification of intrinsic stack pressure in CR2032 coin cells using a pressure-sensitive film. Representative film images (left) and MATLAB-processed pressure maps to quantify the pressure (right) are shown for Ag/Pt@Cu||NMC811 (4 mAh cm^−2^) cells assembled.


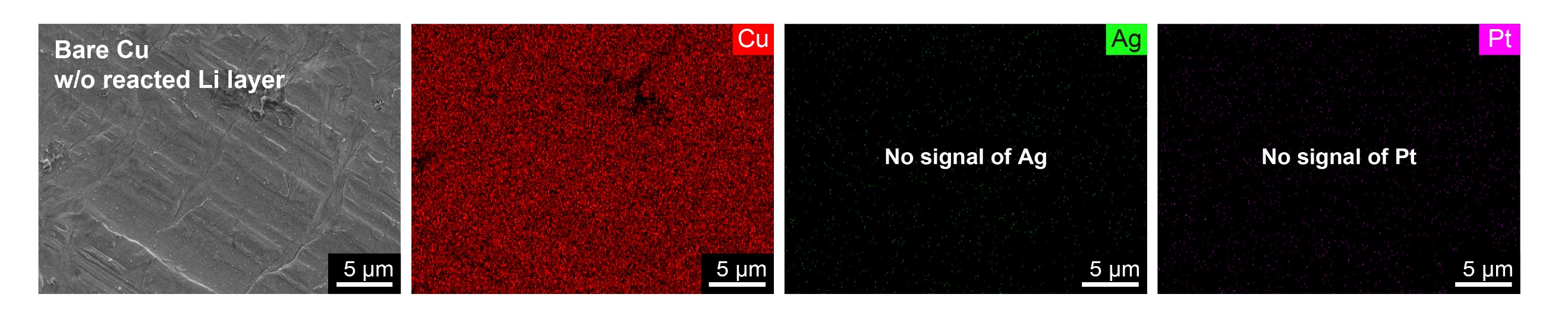


**Figure S17.** Top-view SEM and EDS mappings of bare Cu obtained after removal of the reacted Li layer, prepared by fully stripping the Li reservoir following 20 cycles (SOC-controlled, partial utilization).

**Table S1.** Calculated surface energies of low-Miller-index surfaces of pristine metals (Li, Cu, Ag, and Pt) and selected alloys (LiAg and LiPt).

| System | Index | Surface energy (eV/Å^2^) |
| --- | --- | --- |
| Li | (001) | 0.029 |
|  | (110) | 0.031 |
|  | (111) | 0.033 |
| Cu | (001) | 0.094 |
|  | (110) | 0.097 |
|  | (111) | 0.082 |
| Ag | (001) | 0.052 |
|  | (110) | 0.055 |
|  | (111) | 0.046 |
| Pt | (001) | 0.114 |
|  | (110) | 0.117 |
|  | (111) | 0.092 |
| LiAg | (001)-I | 0.070 |
|  | (001)-II | 0.045 |
|  | (110) | 0.039 |
|  | (111)-I | 0.061 |
|  | (111)-II | 0.044 |
| LiPt | (0001)-I | 0.134 |
|  | (0001)-II | 0.140 |

**Table S2.** Calculated Li adsorption energy ($E_{\mathrm{Li}}^{\mathrm{ads}}$) on various substrate surface structures and corresponding high-symmetry surface sites.

| System | Adsorption site | Adsorption energy  (eV atom^−1^) |
| --- | --- | --- |
| Li | Top | −1.565 |
|  | Bridge | −1.704 |
|  | Hollow | −1.622 |
| Cu | Top | −2.688 |
|  | Bridge | −2.740 |
|  | FCC Hollow | −2.744 |
|  | HCP Hollow | −2.740 |
| Ag | Top | −2.223 |
|  | Bridge | −2.343 |
|  | FCC Hollow | −2.351 |
|  | HCP Hollow | −2.349 |
| Pt | Top | −3.474 |
|  | Bridge | −3.681 |
|  | FCC Hollow | −3.712 |
|  | HCP Hollow | −3.712 |
| LiAg | Top 1 | −2.017 |
|  | Top 2 | −1.726 |
|  | Bridge 1 | −1.863 |
|  | Bridge 2 | −1.897 |
| LiPt | Top 1 | −3.085 |
|  | Bridge | −3.335 |
|  | Hollow 1 | −3.372 |
|  | Hollow 2 | −3.374 |

**Table S3.** Literature comparison of representative protected/modified Cu current collectors for ZE-LMB (Cu||cathode) full cells.

| Ref | Strategy | Cathode | Electrolyte | Electrolyte Amount  / μL | Cathode Areal Capacity  / mAh cm^−2^ | Capacity retention  / % (cycle) |
| --- | --- | --- | --- | --- | --- | --- |
| [8] | Ag@PDA-Cu | NMC111 | 1M LiPF_6_ in EC:DEC | 50 | 2 | 45% (80) |
| [9] | MV-defective carbon | NMC811 | 1M LiPF_6_ EC/DEC + 10 wt% DEC +1 wt% VC | 100 | 4.2 | 90% (50) |
| [10] | BTO-Cu | LCO | 1M LiPF_6_ in EC:DMC | - | 0.8 | 73% (70) |
| [11] | LiF-LiPON Cu | LFP | 1M LiTFSI(DOL/DME) + 2 wt% LiNO_3_ | - | 2 | 66% (100) |
| [12] | GNF-Cu | LFP | 1 M LiTFSI in 1:1 DOL/DME with 1 wt% LiNO_3_ | 40 | 0.45 | 45% (100) |
| [13] | Ag on Cu | NMC811 | 1.0 M LiPF_6_ in an EC/EMC | - | - | 60% (50) |
| [14] | Cu-CNTs | LFP | 1M LiTFSI(DOL/DME) + 3 wt% LiNO_3_ | - | - | 70% (100) |
| [15] | Zr-MG Cu | LFP | 1 M LiTFSI in 1:1 DOL/DME with 2 wt% LiNO_3_ | 70 | 1 | 65% (100) |
| [16] | Curpite-Cu | NMC523 | 6M LiFSI in DME | 30 | 3.3 | 40% (100) |
| [17] | Zn_3_N_2_@Cu | LFP | 1 M LiTFSI in DOL/DME (v/v = 1:1) + 1.0 wt % LiNO_3_ | 66 | 0.57 | 76.20% (50) |
| [18] | FPL-Cu | NMC811 | LiFSI, DME, HFE (1:1.2:2, m:m:m) | 75 | 4 | 71% (100) |
| [19] | Pt-Cu | NCA | 0.6 M LiBF_4_ + 0.6 M LiDFOB in FEC/DEC (1:2=v/v) | 75 | 2.07 | 80% (50) |
| [20] | Zn–AlN@Cu | LFP | 1 M LiTFSI in DOL, DME (v/v=1:1), 1 wt% LiNO_3_ | 60 | 1.6 | 57.93% (100) |
| [21] | Single crystal Cu(111) | NMC811 | 0.6 M LiBF_4_ + 0.6 M LiDFOB in FEC/DEC (1:2=v/v) | 20 | 5.3 | 80% (118) |
| [22] | Cu-Sn@SFPH | NMC523 | 1.5 M LiFSI in DME/TTE (1:4) | 20 | 1.62 | 78.4% (100) |
| [23] | ZnF_2_ on Cu | NMC811 | 1 M LiPF6 in EC, DMC, EMC (v/v/v=1:1:1) | - | 2 | 88% (50) |
| [24] | 2DPA/LN-Cu | LFP | 1-M LiTFSI in 1,3-dioxolane/DME (4:1 in volume) with 3 wt% LiNO_3_ | ~10  (2 μL mAh^−1^) | 3 | 80% (200) |
| [25] | Cu-HEA-F | NMC811 | 1.0 M LiPF_6_ in EC, DEC, and DMC (1:1:1), 10% FEC | 75 | 2 | 80% (35) |
| [26] | AgCu_x_-400 | LFP | 1 M LiPF_6_ in EC/DMC/ DEC (1:1:1, v/v/v) | 60 | 2.16 | 58.8% (100) |
| This work | Ag/Pt@Cu | NMC811 | LiFSI:DME:HFE (1:1.2:3, m/m/m) | 32.4  (6 g Ah^−1^) | 4 | 88% (200) |

**References**

[1] X. Ren, L. Zou, X. Cao, M. H. Engelhard, W. Liu, S. D. Burton, H. Lee, C. Niu, B. E. Matthews, Z. Zhu, C. Wang, B. W. Arey, J. Xiao, J. Liu, J.-G. Zhang, W. Xu, *Joule* **2019**, *3*, 1662.

[2] B. D. Adams, J. Zheng, X. Ren, W. Xu, J.-G. Zhang, *Adv. Energy Mater.* **2018**, *8*, 1702097.

[3] G. Kresse, D. Joubert, *Phys. Rev. B* **1999**, *59*, 1758.

[4] G. Kresse, J. Hafner, *Phys. Rev. B* **1993**, *47*, 558.

[5] G. Kresse, J. Furthmüller, *Phys. Rev. B* **1996**, *54*, 11169.

[6] J. P. Perdew, K. Burke, M. Ernzerhof, *Phys. Rev. Lett.* **1996**, *77*, 3865.

[7] J. Klimeš, D. R. Bowler, A. Michaelides, *J. Phys.: Condens. Matter* **2010**, *22*, 022201.

[8] Z. T. Wondimkun, W. A. Tegegne, J. Shi-Kai, C.-J. Huang, N. A. Sahalie, M. A. Weret, J.-Y. Hsu, P.-L. Hsieh, Y.-S. Huang, S.-H. Wu, W.-N. Su, B. J. Hwang, *Energy Storage Mater.* **2021**, *35*, 334.

[9] H. Kwon, J.-H. Lee, Y. Roh, J. Baek, D. J. Shin, J. K. Yoon, H. J. Ha, J. Y. Kim, H.-T. Kim, *Nat. Commun.* **2021**, *12*, 5537.

[10] C. Wang, M. Liu, M. Thijs, F. G. B. Ooms, S. Ganapathy, M. Wagemaker, *Nat. Commun.* **2021**, *12*, 6536.

[11] J. Sun, S. Zhang, J. Li, B. Xie, J. Ma, S. Dong, G. Cui, *Adv. Mater.* **2023**, *35*, 2209404.

[12] Z. Hou, X. Wang, N. Zhan, Z. Guo, Q. Chen, J. Zhang, T. Bian, B. Hu, Y. Zhao, *Energy Storage Mater.* **2022**, *53*, 254.

[13] W. Shin, A. Manthiram, *ACS Appl. Mater. Interfaces* **2022**, *14*, 17454.

[14] C. Shan, Z. Qin, Y. Xie, X. Meng, J. Chen, Y. Chang, R. Zang, L. Wan, Y. Huang, *Carbon* **2023**, *204*, 367.

[15] J. G. Kim, D. Gu, K.-H. Cho, C.-Y. Im, S. J. Kim, *Small* **2023**, *19*, 2301207.

[16] H.-y. Xia, Y.-k. Wang, Z.-w. Fu, *Appl. Surf. Sci.* **2023**, *617*, 156529.

[17] Y. Zhu, S. Wu, L. Zhang, B. Zhang, B. Liao, *ACS Appl. Mater. Interfaces* **2023**, *15*, 43145.

[18] H.-S. Lim, U. Kim, M. H. Engelhard, D. Kautz, J.-G. Zhang, X. Cao, *Adv. Energy Mater.* **2025**, *15*, 2500778.

[19] J. Seo, J. Lim, H. Chang, J. Lee, J. Woo, I. Jung, Y. Kim, B. Kim, J. Moon, H. Lee, *Small* **2024**, *20*, 2402988.

[20] Y. Zhu, S. Dai, S. Du, B. Zhang, L. Chen, B. Liao, *J. Mater. Chem.* **2024**, *12*, 23622.

[21] M.-H. Kim, D. Y. Kim, Y. Li, J. Kim, M. H. Kim, J. Seo, B. V. Cunning, T. Kim, S.-W. Park, R. S. Ruoff, D.-H. Seo, S. Jin, H.-W. Lee, *Energy Environ. Sci.* **2024**, *17*, 6521.

[22] S. K. Merso, T. M. Tekaligne, M. Adigo Weret, K. N. Shitaw, Y. Nikodimos, S.-C. Yang, Z. B. Muche, B. W. Taklu, B. T. Hotasi, C.-Y. Chang, S.-K. Jiang, G. Brunklaus, M. Winter, S.-H. Wu, W.-N. Su, C.-Y. Mou, B. J. Hwang, *Chem. Eng. J.* **2024**, *485*, 149547.

[23] V. P. Nguyen, H. C. Shim, Y.-W. Byeon, J.-H. Kim, S.-M. Lee, *Adv. Sci.* **2025**, *12*, 2416426.

[24] S. Wang, Y. Wang, Z. Ouyang, S. Geng, Q. Chen, X. Zhao, B. Yuan, X. Zhang, S. Tang, Q. Xu, P. Chen, H. Peng, H. Sun, *Nat. Mater.* **2025**, *24*, 1957.

[25] F. Hu, L. Chen, B. Liao, H. Guo, H. Wang, H. Cao, X. Ouyang, *Energy Storage Mater.* **2025**, *81*, 104497.

[26] H. Yu, P. Sun, H. Cheng, Z. Ding, D. Luo, *Electrochim. Acta* **2025**, *535*, 146623.
